# Supplementary material for: Meta-research: justifying career disruption in funding applications, a survey of Australian researchers
Source: eLife. 2022 Apr 4;11:e76123. doi: 10.7554/eLife.76123 (PMC9038190; doi:10.7554/eLife.76123)
Supplement: Supplementary file 1. [file elife-76123-supp1.docx]

Career disruption - random sample

Start of Block: Consent

| 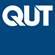 | **PARTICIPANT INFORMATION FOR QUT RESEARCH PROJECT**  **– Survey –** |
| --- | --- |
| **Investigating the use of medical and social circumstances in grant applications: a survey of Australian researchers**  **QUT Ethics Approval Number LR 2021-4303-5402** | |

**Research team**.

| Principal Researcher: | Adrian Barnett (QUT) - Professor of Statistics |
| --- | --- |
| Associate Researchers: | Katie Page (UTS) - Senior Research Fellow  Susanna Cramb (QUT) - Senior Research Fellow |
|  | **School of Public Health and Social Work**  **Queensland University of Technology (QUT)**  **Centre for Health Economics Research and Evaluation**  **University of Technology Sydney (UTS)** |

**Why is the study being conducted?**

This research project is part of our group’s research on research funding. The purpose of this project is to understand what researchers write concerning career disruptions when applying for funding from the National Health and Medical Research Council (NHMRC). You are invited to participate because we believe you are an Australian health and medical researcher. We obtained your name and email from the [*PubMed*](https://pubmed.ncbi.nlm.nih.gov/) database.

**What does participation involve?**

Participation will involve answering 14 to 23 mostly multiple-choice questions that will take approximately 5 to 15 minutes of your time. Questions include:

·    Whether you would include anything on career disruption in an NHMRC application

·    A hypothetical scenario on career disruption

·    Your views on career disruption as a peer reviewer

Your participation in this research is **entirely voluntary**. If you agree to participate you do not have to complete any questions you are uncomfortable answering. Your decision to participate or not participate will in no way impact upon your current or future relationship with QUT. If you do agree to participate you can withdraw from the research project without comment or penalty by emailing a member of the study team (see below). Any information already obtained that can be linked to you will be destroyed.

**What are the possible benefits for me if I take part?**

It is expected that this research project will not directly benefit you. The outcomes of the research, however, may inform national policy on career disruption. The results of the survey and the protocol will be posted here <https://osf.io/m7knu/>.

**What are the possible risks for me if I take part?**

There are no risks beyond normal day-to-day living associated with your participation in this survey. As the questions concern career disruption, there may be some minor discomfort.

QUT provides for limited free psychology, family therapy or counselling services for research participants of QUT research projects who may experience discomfort or distress as a result of their participation in the research. Should you wish to access this service please call the Clinic Receptionist on **07 3138 0999** (Monday–Friday only 9am–5pm), QUT Psychology and Counselling Clinic, 44 Musk Avenue, Kelvin Grove, and indicate that you are a research participant. Alternatively, Lifeline provides access to online, phone or face-to-face support, call **13 11 14** for 24-hour telephone crisis support. If you are aged up to 25, you can also call the Kids Helpline on **1800 551 800**.

**What about privacy and confidentiality?**

Any personal information that could potentially identify you will be removed or changed before files are shared with other researchers or results are made public. The information that will be removed will be your email and any comments you make.

Any data collected as part of this research will be stored securely as per QUT’s Management of research data policy. Data will be stored for a minimum of 5 years, and can be disclosed if it is to protect you or others from harm, if specifically required by law, or if a regulatory or monitoring body such as the ethics committee requests it. When the project is completed, the individual-level data will be published on the Open Science Framework, although none of your text responses will be included.

**How do I give my consent to participate?**

The submission of the survey expresses your consent to participate.

**What if I have questions about the research project?**

If you have any questions or require further information please contact one of the listed researchers:

Adrian Barnett      a.barnett@qut.edu.au      07 3138 6010

Susanna Cramb    susanna.cramb@qut.edu.au  07 3138 6213

**What if I have a concern or complaint regarding the conduct of the research project?**

QUT is committed to research integrity and the ethical conduct of research projects.  If you wish to discuss the study with someone not directly involved, particularly in relation to matters concerning policies, information or complaints about the conduct of the study or your rights as a participant, you may contact the QUT Research Ethics Advisory Team on 07 3138 5123 or email [humanethics@qut.edu.au](mailto:humanethics@qut.edu.au).

**Thank you for helping with this research project. Please**[**download**](https://mfr.au-1.osf.io/render?url=https://osf.io/r4b8y/?direct%26mode=render%26action=download%26mode=render)**this sheet for your information.**

I consent to participate

- Yes

End of Block: Consent

Start of Block: Your NHMRC activity in the last five years

| 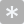 |
| --- |

How many NHMRC applications have you been part of in the last 5 years as a chief investigator? Please include all types, so Projects Grants, Fellowships, Partnerships, Centres of Research Excellence, etc. Include all applications, whether funded or not.

________________________________________________________________

How often in those applications did you include medical or social circumstance that disrupted your research?

- Always
- Very Often
- Sometimes
- Rarely
- Never

End of Block: Your NHMRC activity in the last five years

Start of Block: Knowledge of current NHMRC policies

How aware are you of the current NHMRC policies around documenting career disruption in grant applications?

- I don’t know any NHMRC documents or policies on career disruption
- I am aware of some of the NHMRC documents or policies on career disruption
- I am fully aware of all relevant NHMRC documents and policies on career disruption

I understand what medical or social circumstances could be mentioned in an NHMRC career disruption section.

- Disagree
- Neutral
- Agree

Do you have any comments on this section? (Optional)

________________________________________________________________

________________________________________________________________

________________________________________________________________

________________________________________________________________

________________________________________________________________

End of Block: Knowledge of current NHMRC policies

Start of Block: Current NHMRC applications

In this section, please answer as if you were completing an NHMRC application today. This could be any application (Fellowship, Project, Centre, etc) where you are a chief investigator and hence could include medical or social circumstances. We are NOT asking for details about your personal circumstances, but rather want to know what details you would include.

Would you write anything in your application about disruption to your research because of medical or social circumstances in the last five years?

- No, I have no medical or social circumstances to write about
- No, I have medical or social circumstances but would not include them
- Yes, I have medical or social circumstances and would include them
- Other

Other, please specify

________________________________________________________________

________________________________________________________________

________________________________________________________________

________________________________________________________________

________________________________________________________________

Considering medical or social circumstances you would not include, is this because

|  | Agree | Neutral | Disagree | Not applicable |
| --- | --- | --- | --- | --- |
| My circumstances have had only a minor impact on my research |  |  |  |  |
| I am concerned that sharing this information would reduce my chances of winning funding |  |  |  |  |
| I do not want to share personal details with my co-investigators |  |  |  |  |
| I do not want to share personal details with peer reviewers |  |  |  |  |
| I am worried about my reputation |  |  |  |  |
| I am unsure of how to use the “career disruption” and/or “career context” sections in NHMRC applications |  |  |  |  |
| Career disruption is just part of the lottery of life and should not be adjusted for |  |  |  |  |

Any other thoughts around medical or social circumstances you would not include (Optional)

________________________________________________________________

________________________________________________________________

________________________________________________________________

________________________________________________________________

________________________________________________________________

Considering medical or social circumstances you would include, would you

|  | Agree | Neutral | Disagree | Not applicable |
| --- | --- | --- | --- | --- |
| Share personal details about the issue |  |  |  |  |
| Share only the basic information needed to convey the issue |  |  |  |  |
| Not share any details about the issue, but instead state how much time you’ve lost to research |  |  |  |  |

Any other thoughts around medical or social circumstances you would include (Optional)

________________________________________________________________

________________________________________________________________

________________________________________________________________

________________________________________________________________

________________________________________________________________

Do you have any comments on this section? (Optional)

________________________________________________________________

________________________________________________________________

________________________________________________________________

________________________________________________________________

________________________________________________________________

End of Block: Current NHMRC applications

Start of Block: Hypothetical NHMRC application – respondents received one of the following four scenarios at random.

In this section, please consider the following hypothetical scenario and think about what you would write in the career disruption section.

Imagine you had missed six months of full-time research in the previous year because of caring for an elderly relative. You had no other personal or medical circumstances in the last five years.

Would you write anything in the career disruption section about the six months spent away from research because of this issue?

- Yes
- No

In this section, please consider the following hypothetical scenario and think about what you would write in the career disruption section. Imagine you had missed six months of full-time research in the previous year because of caring for a child. You had no other personal or medical circumstances in the last five years. Would you write anything in the career disruption section about the six months spent away from research because of this issue?

- Yes
- No

In this section, please consider the following hypothetical scenario and think about what you would write in the career disruption section.

Imagine you had missed six months of full-time research in the previous year because of a car accident that led to two months in hospital. You had no other personal or medical circumstances in the last five years.

Would you write anything in the career disruption section about the six months spent away from research because of this issue?

- Yes
- No

In this section, please consider the following hypothetical scenario and think about what you would write in the career disruption section.

Imagine you had missed six months of full-time research in the previous year because of severe depression that included two months in rehabilitation. You had no other personal or medical circumstances in the last five years.

Would you write anything in the career disruption section about the six months spent away from research because of this issue?

- Yes
- No

End of Block: Hypothetical NHMRC application

Start of Block: Hypothetical NHMRC application - follow-up

Would you not write about this career disruption because

|  | Agree | Neutral | Disagree |
| --- | --- | --- | --- |
| This issue should only have a minor impact on my research |  |  |  |
| Because sharing this information could reduce my chances of winning funding |  |  |  |
| I would not want to share personal details with my co-investigators |  |  |  |
| I would not want to share personal details with my peer reviewers |  |  |  |
| I would be worried about my reputation |  |  |  |
| I am unsure of how to use the “career disruption” and/or “career context” sections in NHMRC applications. |  |  |  |
| Career disruption is just part of the lottery of life and should not be adjusted for. |  |  |  |

Any other thoughts around what you would not include (Optional)

________________________________________________________________

________________________________________________________________

________________________________________________________________

________________________________________________________________

________________________________________________________________

Would you

|  | Agree | Neutral | Disagree |
| --- | --- | --- | --- |
| Share all my personal details about the issue |  |  |  |
| Partially share my personal details about the issue |  |  |  |
| Not share any personal details |  |  |  |

Any other thoughts around what you would include (Optional)

________________________________________________________________

________________________________________________________________

________________________________________________________________

________________________________________________________________

________________________________________________________________

How much time away from research would you include in your career disruption section because of this issue?

|  | 0 | 2 | 4 | 6 | 8 | 10 | 12 |
| --- | --- | --- | --- | --- | --- | --- | --- |

| Months | 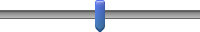 |
| --- | --- |

Do you have any comments on this section? (Optional)

________________________________________________________________

________________________________________________________________

________________________________________________________________

________________________________________________________________

________________________________________________________________

End of Block: Hypothetical NHMRC application - follow-up

Start of Block: Peer Reviewer

Now imagine you are a grant peer reviewer instead of an applicant.

Imagine you are comparing two NHMRC fellowship applications for which both applicants have stated that they lost 6 months of research time in the last 5 years due to career disruption. One applicant does not give any details and the other explains their medical issue in detail.

When adjusting the two applicants’ track records for career disruption, how much time would you adjust for for each applicant

|  | 0 | 2 | 4 | 6 | 8 | 10 | 12 |
| --- | --- | --- | --- | --- | --- | --- | --- |

| Applicant who gave no details | 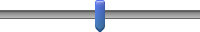 |
| --- | --- |
| Applicant who explained their medical issue in detail | 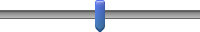 |

Imagine you are reading a career disruption section about somebody that you know. This is not a close colleague, as conflict of interest rules would mean you could not review their application.

How comfortable are you reading personal social and/or medical details as part of the peer review process?

- Extremely comfortable
- Somewhat comfortable
- Neither comfortable nor uncomfortable
- Somewhat uncomfortable
- Extremely uncomfortable

Do you have any comments on this section? (Optional)

________________________________________________________________

________________________________________________________________

________________________________________________________________

________________________________________________________________

________________________________________________________________

End of Block: Peer Reviewer

Start of Block: Independent panel for assessing career disruption

Career disruption sections are currently assessed by scientific panels, who judge how a personal or medical issue has impacted on the researcher’s track record. An alternative is an independent panel of medical and psychological experts who would read applicants’ career disruption sections that detailed their medical and/or social circumstances. The expert panel would then write a short report, such as “This applicant has lost the equivalent of 6 months of full-time work in the last 5 years”. The scientific panel would see this short report and not the detailed career disruption section. The scientific panel would then make their adjustment to the researcher’s track record. This could allow researchers to confidentially share detailed medical and personal information.

An independent medical panel would increase administrative costs and could delay the peer review process.

How do you think career disruption sections in NHMRC applications should be assessed?

- Scientific panel (current system)
- Independent medical panel (new system)
- Not sure
- Other

Please specify

________________________________________________________________

Do you have any comments on this section? (Optional)

________________________________________________________________

________________________________________________________________

________________________________________________________________

________________________________________________________________

________________________________________________________________

End of Block: Independent panel for assessing career disruption

Start of Block: Demographics

Lastly, we'd like to know some basic information about you.

What is your broad research area?

- Basic Science
- Clinical Medicine and Science
- Public Health
- Health Services Research

Gender

- Male
- Female
- Non-binary / third gender
- I use a different term (please specify)
- Prefer not to say

Approximate number of years in health and medical research. Answer in terms of working years, e.g., if 6 years of working half-time then answer 3 years ("5 years or less").

- 5 years or less
- 6 to 10 years
- 11 to 15 years
- 16 to 20 years
- 21 years or more

Do you have any comments on this survey? (Optional)

________________________________________________________________

________________________________________________________________

________________________________________________________________

________________________________________________________________

________________________________________________________________

End of Block: Demographics
