# Supplementary material for: Meta-research: justifying career disruption in funding applications, a survey of Australian researchers
Source: eLife. 2022 Apr 4;11:e76123. doi: 10.7554/eLife.76123 (PMC9038190; doi:10.7554/eLife.76123)
Supplement: Supplementary file 2. [file elife-76123-supp2.docx]

Career disruption survey: summary results

Adrian Barnett

24 March 2022

Table of Contents

## Introduction

All the results are stratified by the two samples: random and non-random. The total number of responses was 246, with 124 from the random sample and 122 from the non-random sample.

Text *in italics* is the direct wording from the survey

# *Your NHMRC activity in the last five years*

## *How many NHMRC applications have you been part of in the last 5 years as a chief investigator?*

| **sample** | **Median (IQR)** |
| --- | --- |
| Non-random | 5 (3 to 10) |
| Random | 2 (0 to 6) |
| Total | 4 (1 to 8) |

Cells show the median and inter-quartile range.


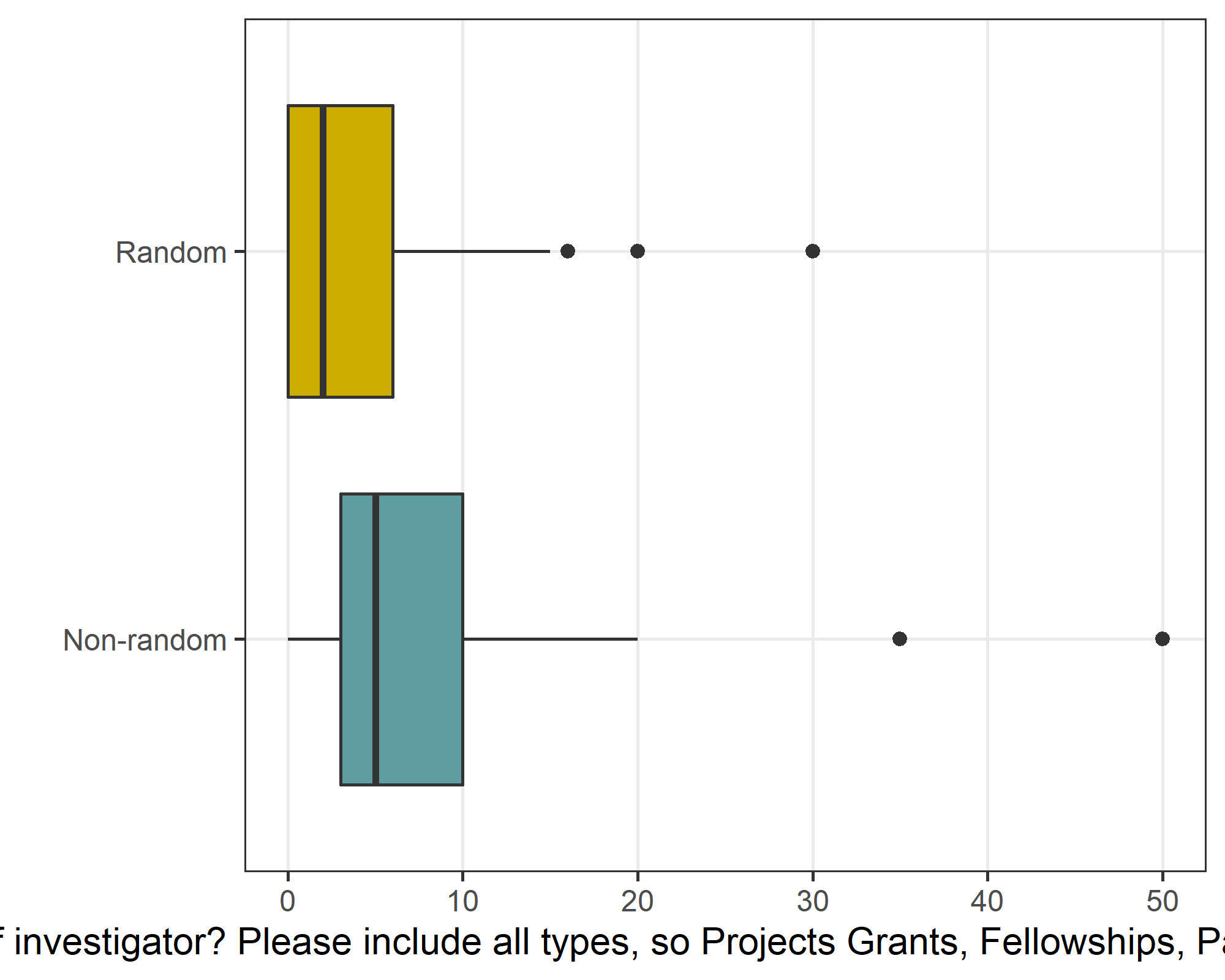


The few results over 30 are outliers and are 6 per year. It is possible that these respondent included applications where they were an associate investigator and/or ARC applications.

## *How often in those applications did you include medical or social circumstance that disrupted your research?*

The results below are only for people who had at least 1 application.

| **Response** | **Non-random** | **Random** | **Total** |
| --- | --- | --- | --- |
| Always | 53 (44) | 13 (15) | 66 (32) |
| Very Often | 12 (10) | 5 (6) | 17 (8) |
| Sometimes | 15 (12) | 13 (15) | 28 (14) |
| Rarely | 8 (7) | 12 (14) | 20 (10) |
| Never | 32 (27) | 44 (51) | 76 (37) |


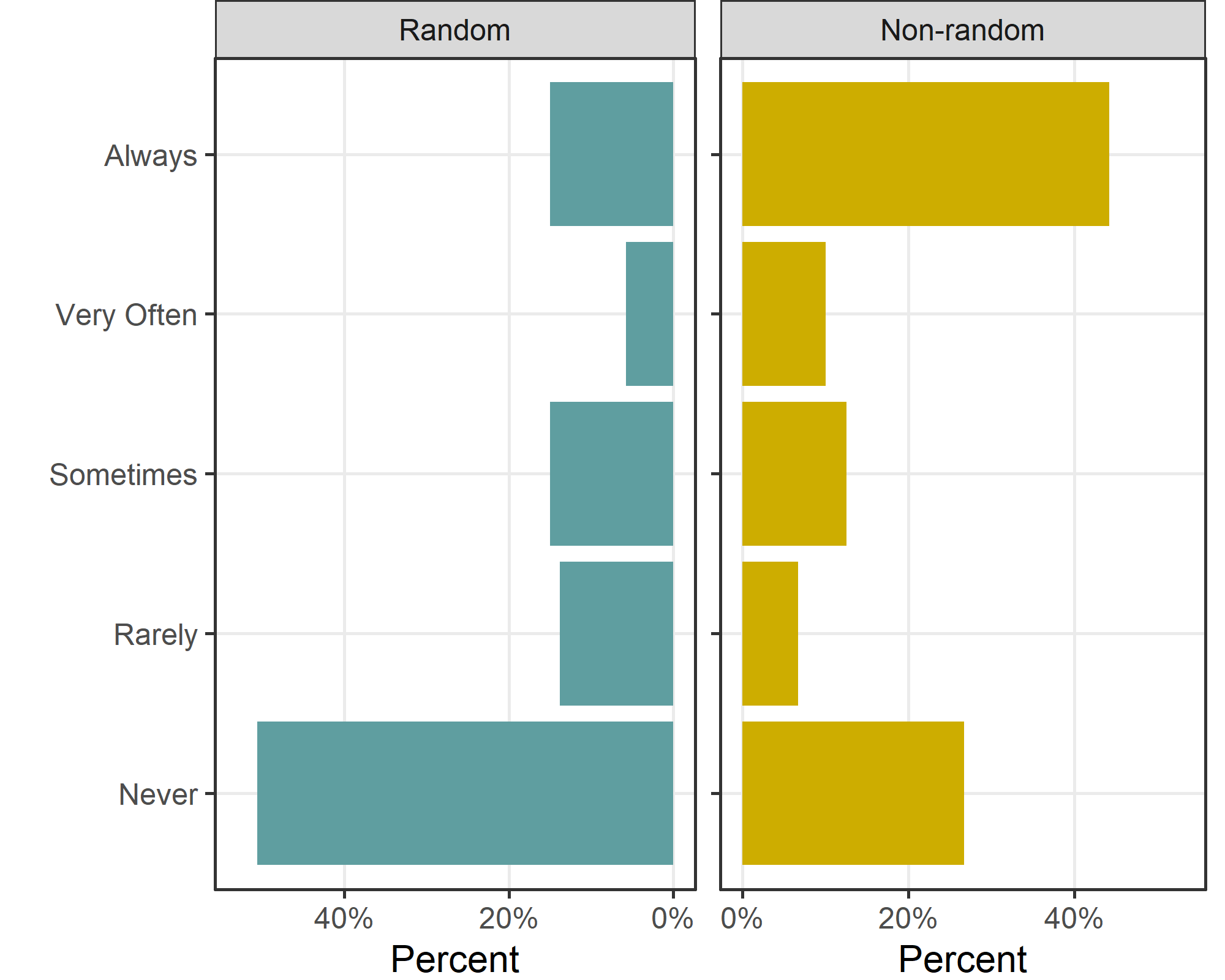


##### Confidence intervals for random sample:

We give confidence intervals for the random sample in order to estimate the prevalence in the wider population.

| **Response** | **n** | **p** | **lower** | **upper** |
| --- | --- | --- | --- | --- |
| Always | 13 | 15 | 6 | 26 |
| Very Often | 5 | 6 | 0 | 17 |
| Sometimes | 13 | 15 | 6 | 26 |
| Rarely | 12 | 14 | 5 | 25 |
| Never | 44 | 51 | 41 | 62 |

# Knowledge of current NHMRC policies

## *How aware are you of the current NHMRC policies around documenting career disruption in grant applications?*

| **Response** | **Non-random** | **Random** | **Total** |
| --- | --- | --- | --- |
| I don’t know any NHMRC documents or policies on career disruption | 8 (7) | 33 (27) | 41 (17) |
| I am aware of some of the NHMRC documents or policies on career disruption | 65 (53) | 67 (55) | 132 (54) |
| I am fully aware of all relevant NHMRC documents and policies on career disruption | 49 (40) | 22 (18) | 71 (29) |

##### Confidence intervals for awareness

| **Response** | **n** | **p** | **lower** | **upper** |
| --- | --- | --- | --- | --- |
| I don’t know any NHMRC documents or policies on career disruption | 33 | 27 | 19 | 37 |
| I am aware of some of the NHMRC documents or policies on career disruption | 67 | 55 | 47 | 65 |
| I am fully aware of all relevant NHMRC documents and policies on career disruption | 22 | 18 | 10 | 28 |

## *I understand what medical or social circumstances could be mentioned in an NHMRC career disruption section*

| **Response** | **Non-random** | **Random** | **Total** |
| --- | --- | --- | --- |
| Disagree | 7 (6) | 26 (21) | 33 (14) |
| Neutral | 25 (20) | 40 (33) | 65 (27) |
| Agree | 90 (74) | 56 (46) | 146 (60) |


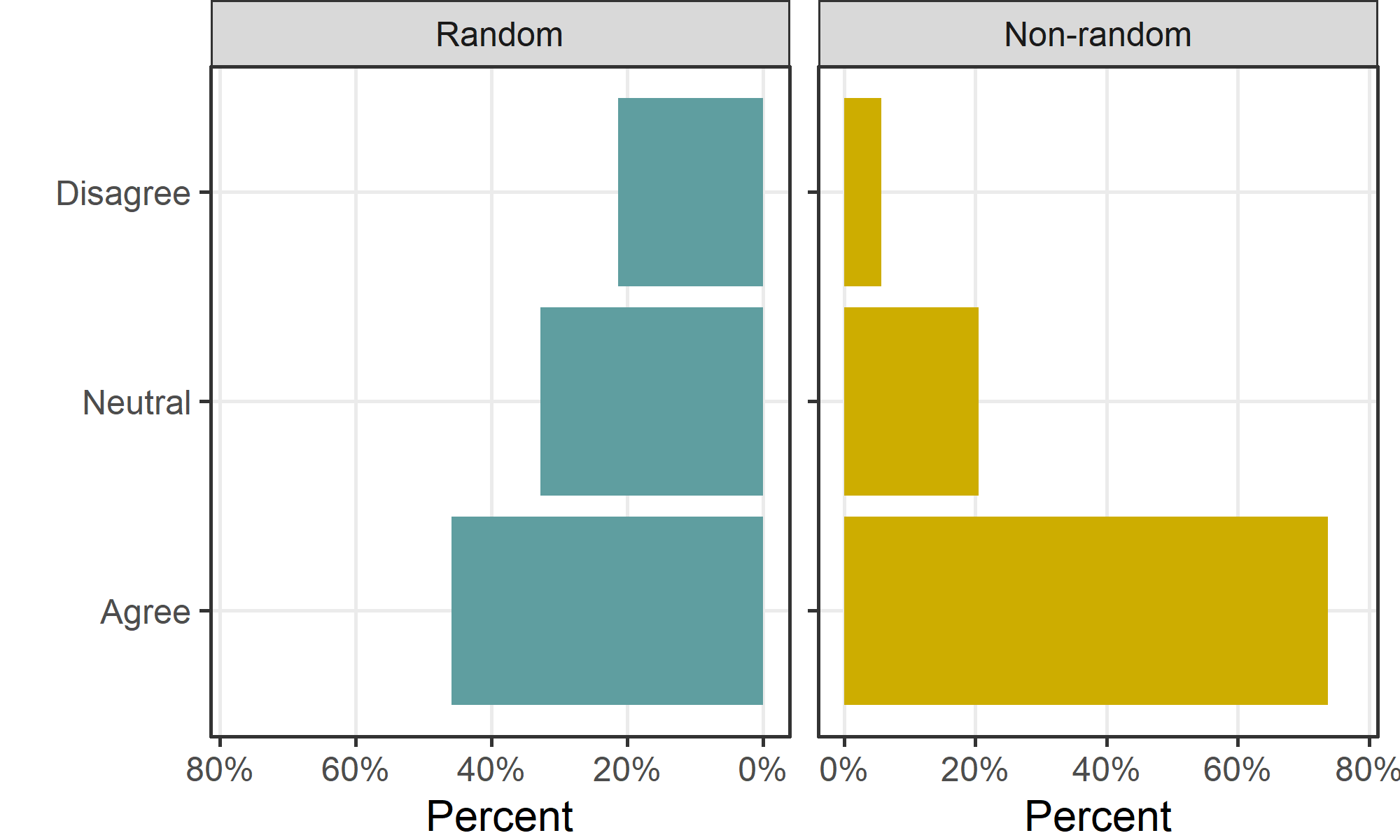


The non-random sample are more informed about the policy, likely because they have had to read up on them.

# Current NHMRC applications

## *Would you write anything in your application about disruption to your research because of medical or social circumstances in the last five years?*

| **Response** | **Non-random** | **Random** | **Total** |
| --- | --- | --- | --- |
| No, I have no medical or social circumstances to write about | 27 (22) | 57 (47) | 84 (35) |
| No, I have medical or social circumstances but would not include them | 15 (12) | 16 (13) | 31 (13) |
| Yes, I have medical or social circumstances and would include them | 78 (64) | 42 (35) | 120 (50) |
| Other | 1 (1) | 6 (5) | 7 (3) |

The numbers above show that career disruption applies to a relatively large number of applicants. The non-random sample were much more likely to have relevant circumstances, and they were likely motivated to complete the survey because of their experiences.


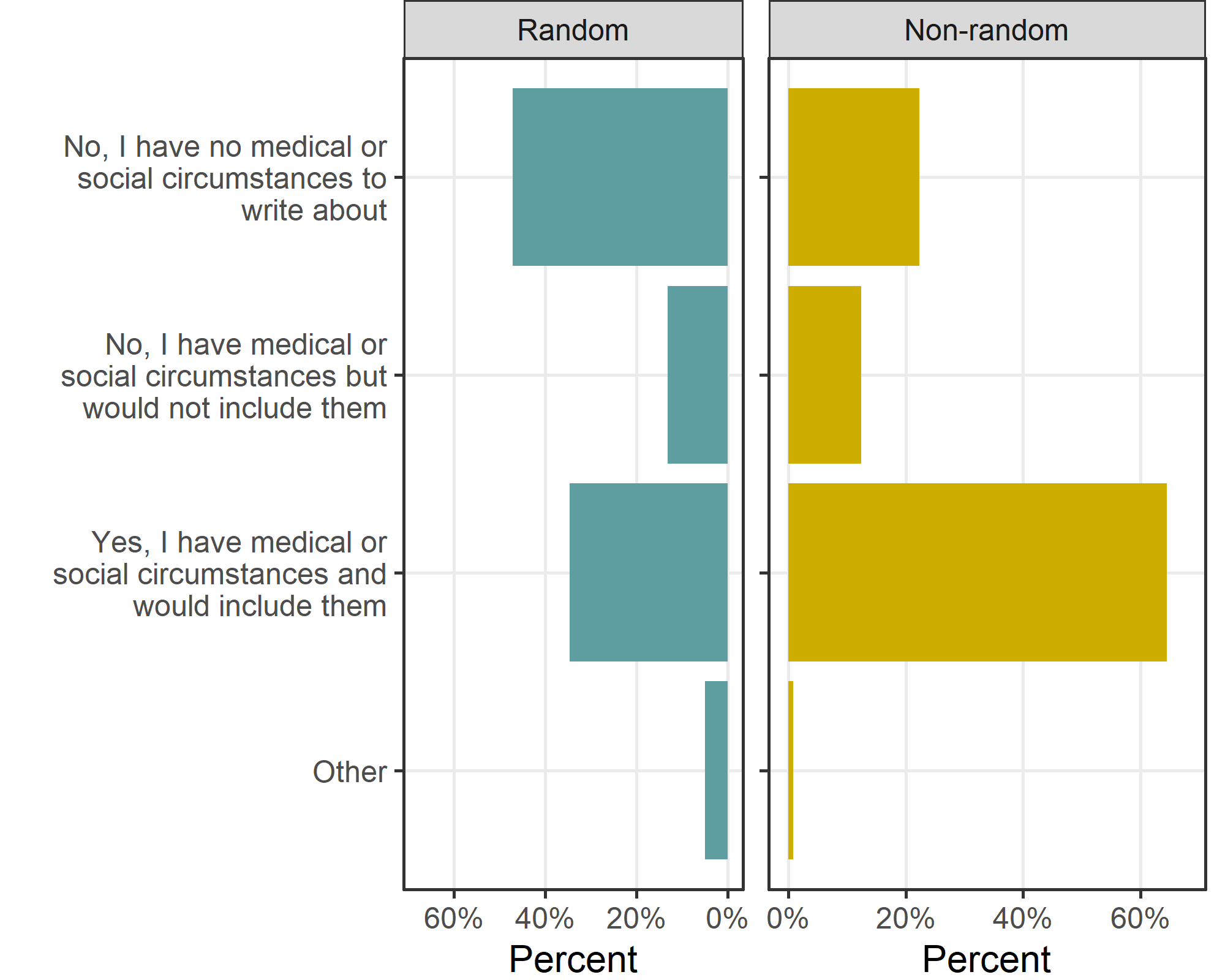


##### Confidence intervals for random sample:

| **Response** | **n** | **p** | **lower** | **upper** |
| --- | --- | --- | --- | --- |
| No, I have no medical or social circumstances to write about | 57 | 47 | 38 | 56 |
| No, I have medical or social circumstances but would not include them | 16 | 13 | 4 | 23 |
| Yes, I have medical or social circumstances and would include them | 42 | 35 | 26 | 44 |
| Other | 6 | 5 | 0 | 14 |

## *Considering medical or social circumstances you would not include, is this because*

The results below are ordered by the overall number of ‘Agree’ responses.

| **Question** | **Response** | **Non-random** | **Random** |
| --- | --- | --- | --- |
| My circumstances have had only a minor impact on my research | Agree | 33 (40) | 47 (52) |
|  | Neutral | 9 (11) | 12 (13) |
|  | Disagree | 41 (49) | 31 (34) |
| I am concerned that sharing this information would reduce my chances of winning funding | Agree | 41 (45) | 30 (32) |
|  | Neutral | 16 (17) | 30 (32) |
|  | Disagree | 35 (38) | 35 (37) |
| I am worried about my reputation | Agree | 39 (41) | 28 (30) |
|  | Neutral | 14 (15) | 18 (19) |
|  | Disagree | 41 (44) | 47 (51) |
| I do not want to share personal details with peer reviewers | Agree | 34 (37) | 32 (33) |
|  | Neutral | 16 (17) | 19 (20) |
|  | Disagree | 42 (46) | 45 (47) |
| I do not want to share personal details with my co-investigators | Agree | 32 (35) | 24 (25) |
|  | Neutral | 8 (9) | 16 (17) |
|  | Disagree | 51 (56) | 56 (58) |
| I am unsure of how to use the “career disruption” and/or “career context” sections in NHMRC applications | Agree | 24 (24) | 30 (32) |
|  | Neutral | 24 (24) | 24 (26) |
|  | Disagree | 50 (51) | 39 (42) |
| Career disruption is just part of the lottery of life and should not be adjusted for | Agree | 2 (2) | 6 (6) |
|  | Neutral | 6 (6) | 13 (13) |
|  | Disagree | 90 (92) | 80 (81) |


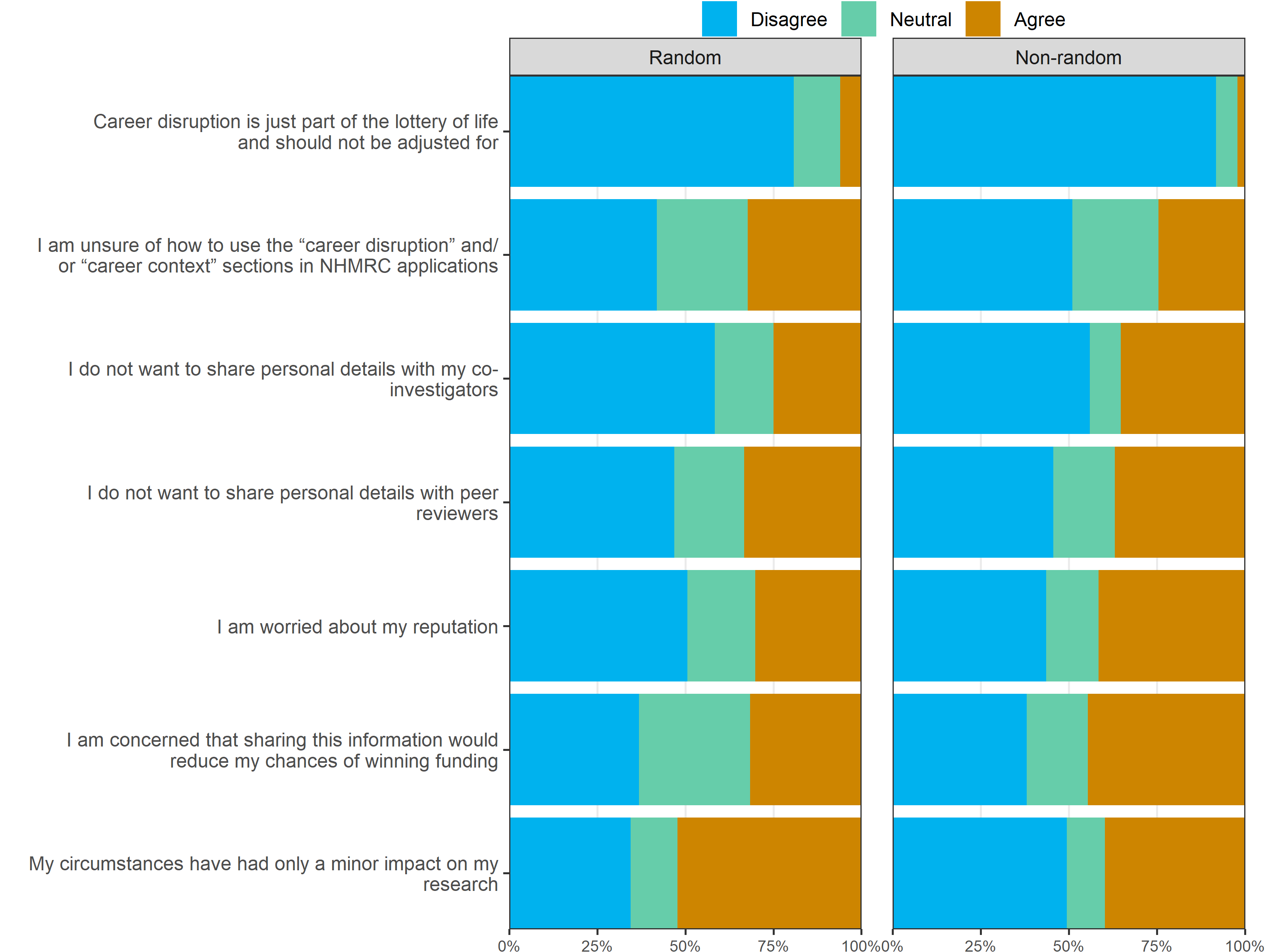


## *Considering medical or social circumstances you would include, would you*

The results below are ordered by the overall number of ‘Agree’ responses.

| **Question** | **Response** | **Non-random** | **Random** |
| --- | --- | --- | --- |
| Share only the basic information needed to convey the issue | Agree | 91 (83) | 85 (80) |
|  | Neutral | 10 (9) | 15 (14) |
|  | Disagree | 8 (7) | 6 (6) |
| Not share any details about the issue, but instead state how much time you’ve lost to research | Agree | 36 (34) | 32 (31) |
|  | Neutral | 29 (27) | 30 (29) |
|  | Disagree | 41 (39) | 40 (39) |
| Share personal details about the issue | Agree | 33 (31) | 21 (21) |
|  | Neutral | 42 (40) | 39 (38) |
|  | Disagree | 31 (29) | 42 (41) |


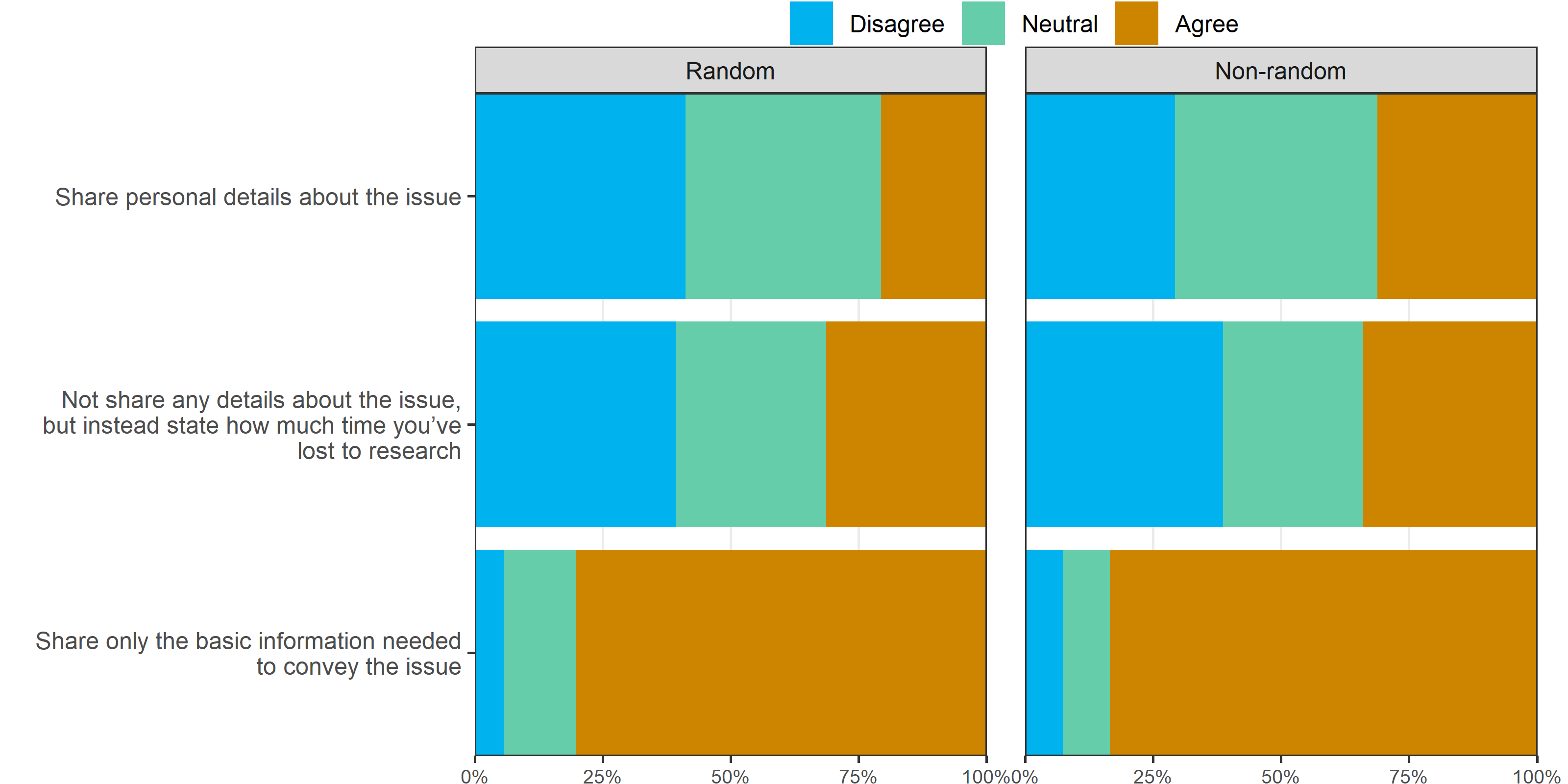


# Hypothetical NHMRC application

*In this section, please consider the following hypothetical scenario and think about what you would write in the career disruption section*

*Imagine you had missed six months of full-time research in the previous year because [one of 4 random scenarios]. You had no other personal or medical circumstances in the last five years.*

## *Would you write anything in the career disruption section about the six months spent away from research because of this issue?*

| **sample** | **Scenario** | **No** | **Yes** |
| --- | --- | --- | --- |
| Non-random | A car accident | 1 (4) | 25 (96) |
|  | Caring for a child | 1 (3) | 28 (97) |
|  | Caring for an elderly relative | 5 (16) | 26 (84) |
|  | Severe depression | 4 (14) | 24 (86) |
| Random | A car accident | 4 (14) | 25 (86) |
|  | Caring for a child | 6 (18) | 27 (82) |
|  | Caring for an elderly relative | 5 (15) | 29 (85) |
|  | Severe depression | 7 (33) | 14 (67) |


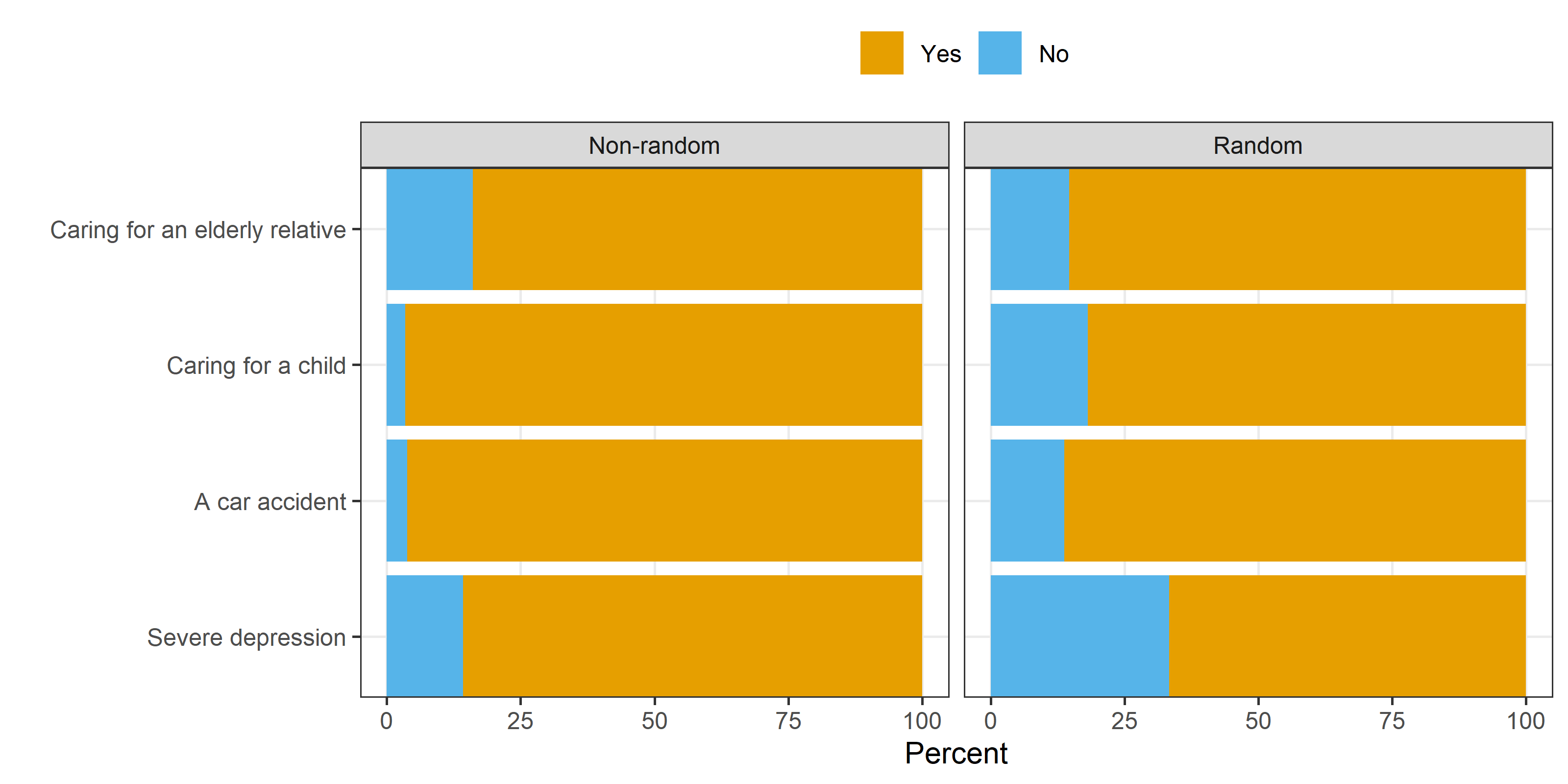


The plot rows are ordered by the overall frequency of “Yes”.

## *Would you not write about this career disruption because*

The following table is only for people who answered “No” to writing any career disruption.

| **Question** | **Response** | **Non-random** | **Random** |
| --- | --- | --- | --- |
| I would not want to share personal details with my peer reviewers | Agree | 8 (80) | 13 (65) |
|  | Neutral | 2 (20) | 3 (15) |
|  | Disagree | 0 | 4 (20) |
| Because sharing this information could reduce my chances of winning funding | Agree | 9 (90) | 11 (55) |
|  | Neutral | 1 (10) | 4 (20) |
|  | Disagree | 0 | 5 (25) |
| I would not want to share personal details with my co-investigators | Agree | 7 (70) | 12 (60) |
|  | Neutral | 2 (20) | 1 (5) |
|  | Disagree | 1 (10) | 7 (35) |
| I would be worried about my reputation | Agree | 8 (89) | 11 (55) |
|  | Neutral | 1 (11) | 3 (15) |
|  | Disagree | 0 | 6 (30) |
| I am unsure of how to use the “career disruption” and/or “career context” sections in NHMRC applications. | Agree | 5 (50) | 6 (30) |
|  | Neutral | 1 (10) | 5 (25) |
|  | Disagree | 4 (40) | 9 (45) |
| This issue should only have a minor impact on my research | Agree | 0 | 6 (30) |
|  | Neutral | 1 (10) | 1 (5) |
|  | Disagree | 9 (90) | 13 (65) |
| Career disruption is just part of the lottery of life and should not be adjusted for. | Agree | 0 | 2 (10) |
|  | Neutral | 2 (20) | 3 (15) |
|  | Disagree | 8 (80) | 15 (75) |

## *Would you*

The following table is only for people who answered “Yes” to writing any career disruption.

| **Question** | **Response** | **Non-random** | **Random** |
| --- | --- | --- | --- |
| Partially share my personal details about the issue | Agree | 69 (70) | 61 (66) |
|  | Neutral | 15 (15) | 16 (17) |
|  | Disagree | 15 (15) | 16 (17) |
| Share all my personal details about the issue | Agree | 31 (31) | 21 (22) |
|  | Neutral | 28 (28) | 33 (35) |
|  | Disagree | 40 (40) | 41 (43) |
| Not share any personal details | Agree | 8 (8) | 12 (13) |
|  | Neutral | 20 (20) | 16 (18) |
|  | Disagree | 70 (71) | 63 (69) |

## *How much time away from research would you include in your career disruption section because of this issue?*

The following table is only for people who answered “Yes” to writing any career disruption.

| **Scenario** | **Non-random** | **Random** |
| --- | --- | --- |
| A car accident | 6.0 (6.0 to 9.9) | 6.0 (4.0 to 7.0) |
| Caring for a child | 6.0 (6.0 to 6.0) | 6.0 (6.0 to 9.7) |
| Caring for an elderly relative | 6.0 (5.5 to 8.0) | 6.0 (3.9 to 6.0) |
| Severe depression | 6.0 (5.0 to 6.0) | 6.0 (5.9 to 10.5) |

The cells show the median and inter-quartile range.


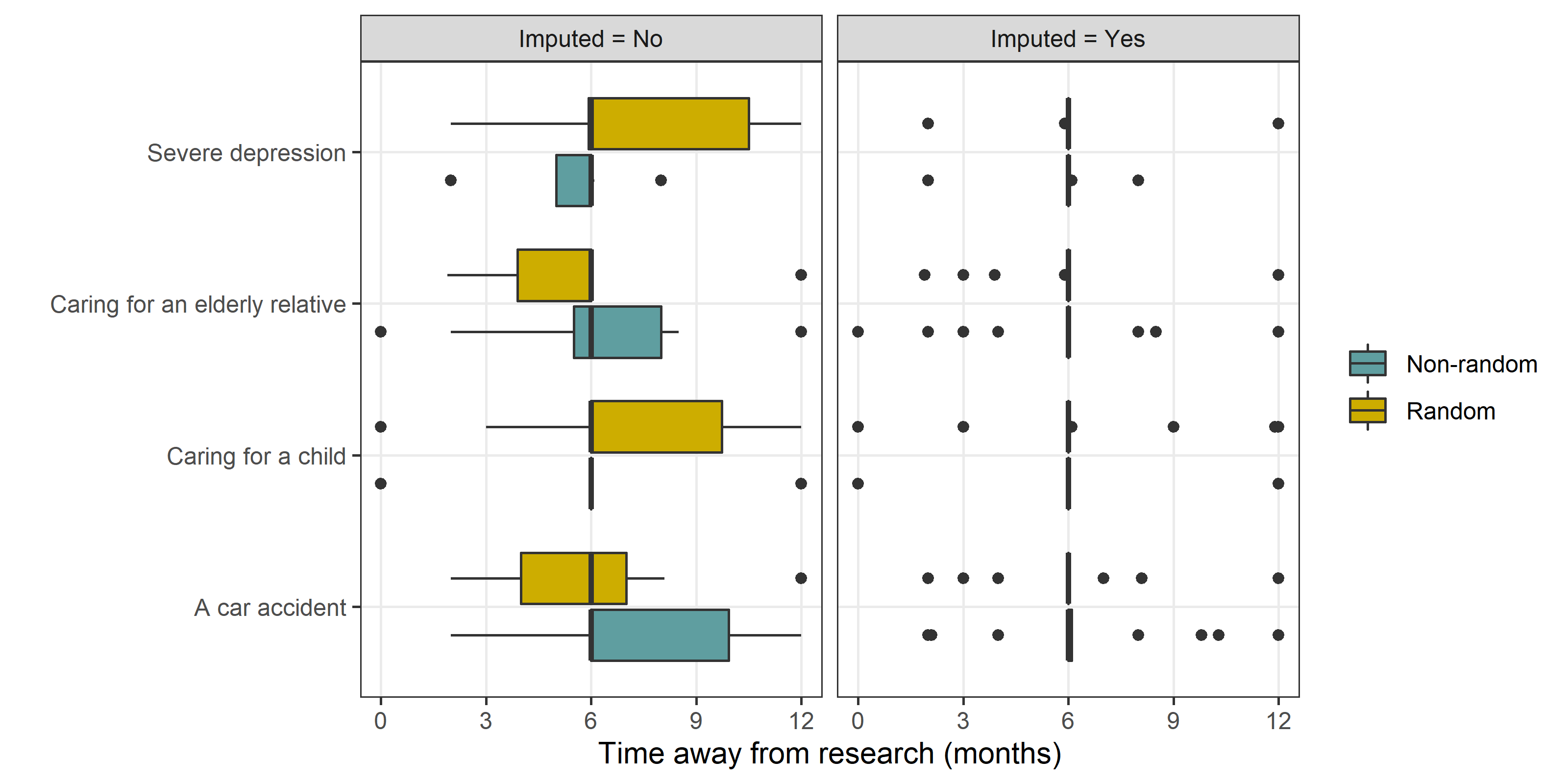


The two scenarios of “caring for an elderly relative” and “severe depression” generally have the lowest times.

#### Estimated means by scenario

The plot below shows the estimated means from a Bayesian regression model that included the scenario and the sample (random/non-random) as predictors. The plot shows the mean times and 95% credible intervals. The numbers below the means are the estimated probability that the scenario has the lowest mean. There’s a high probability (0.80) that severe depression has the smallest average time adjusted, and a low probability that caring for a child or elderly relative has the smallest adjustment.


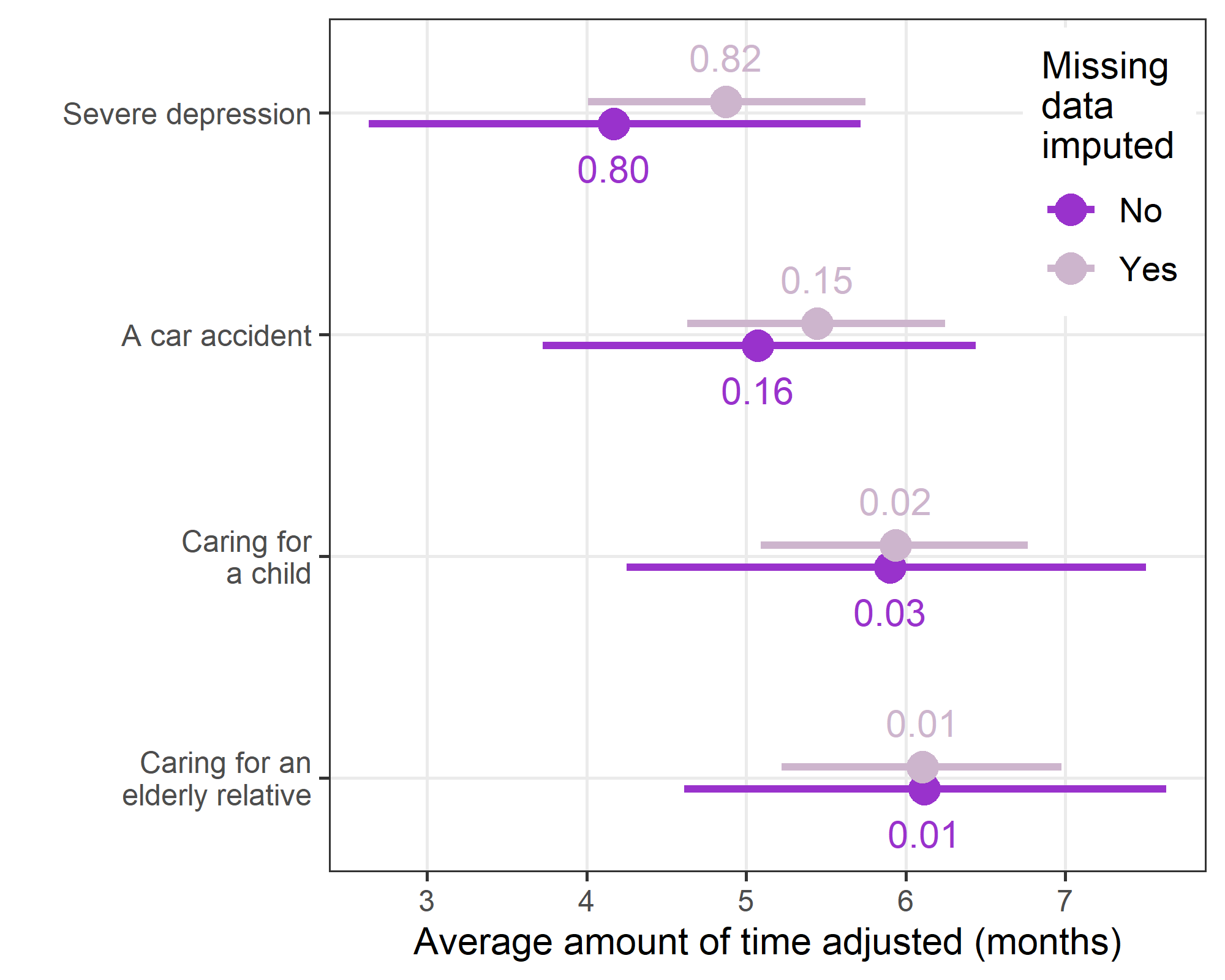


# Peer reviewer section

*Now imagine you are a grant peer reviewer instead of an applicant.*

## Adjusting track record

*Imagine you are comparing two NHMRC fellowship applications for which both applicants have stated that they lost 6 months of research time in the last 5 years due to career disruption. One applicant does not give any details and the other explains their medical issue in detail*.

The plot below shows the times that respondents gave for the two scenarios.


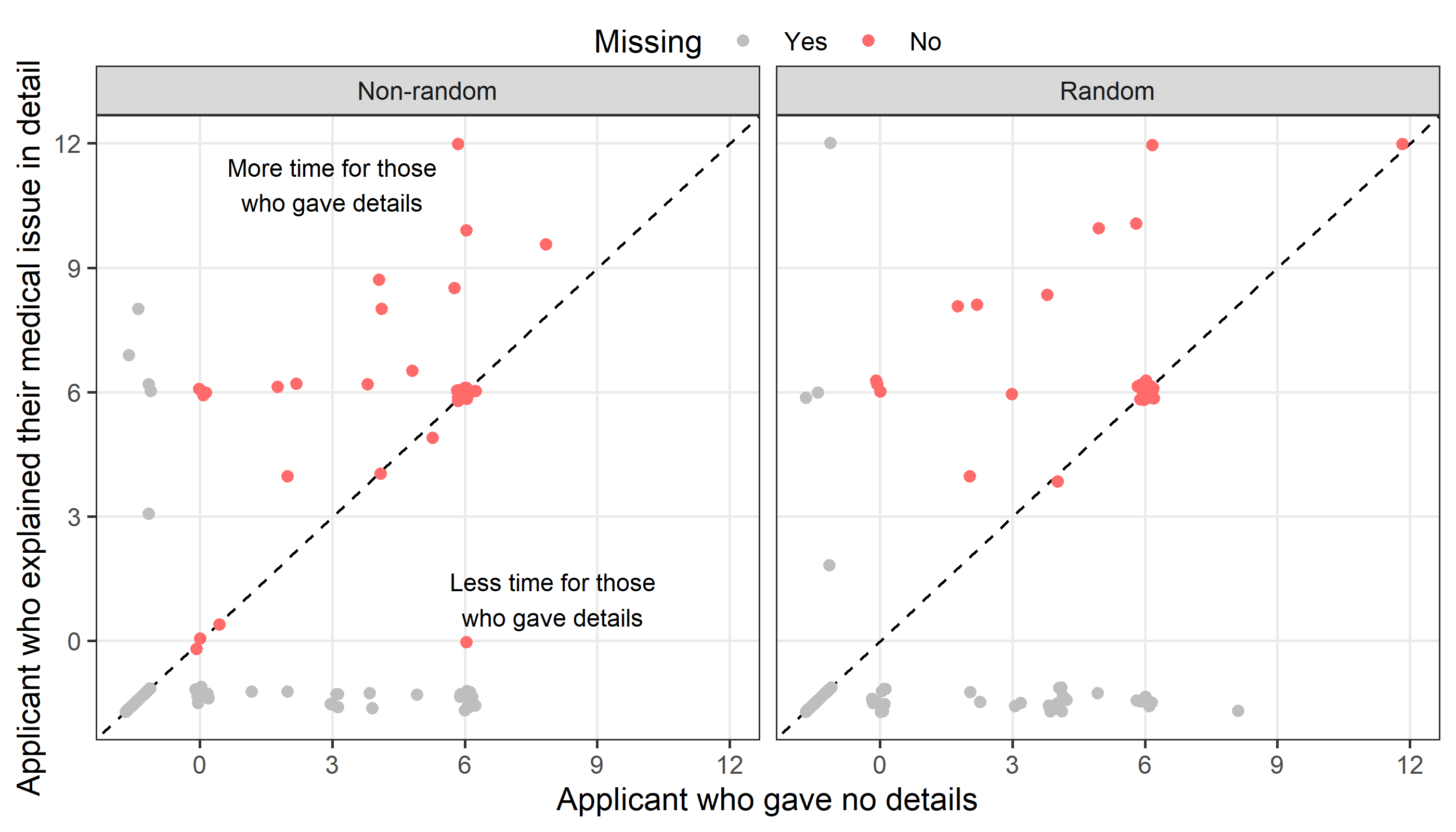


The points have been jittered to avoid overlap as lots of people gave round numbers. The diagonal dotted line shows perfect agreement for the two times.

The plot includes the missing results, where either both sliders were missing (dots in bottom-left) or just one slider was missing (dots along bottom or left of the plot).

## Paired difference within respondents

We examine the paired differences within respondents for the time adjustment using the difference of the applicant who gave details from the applicant who gave no details.


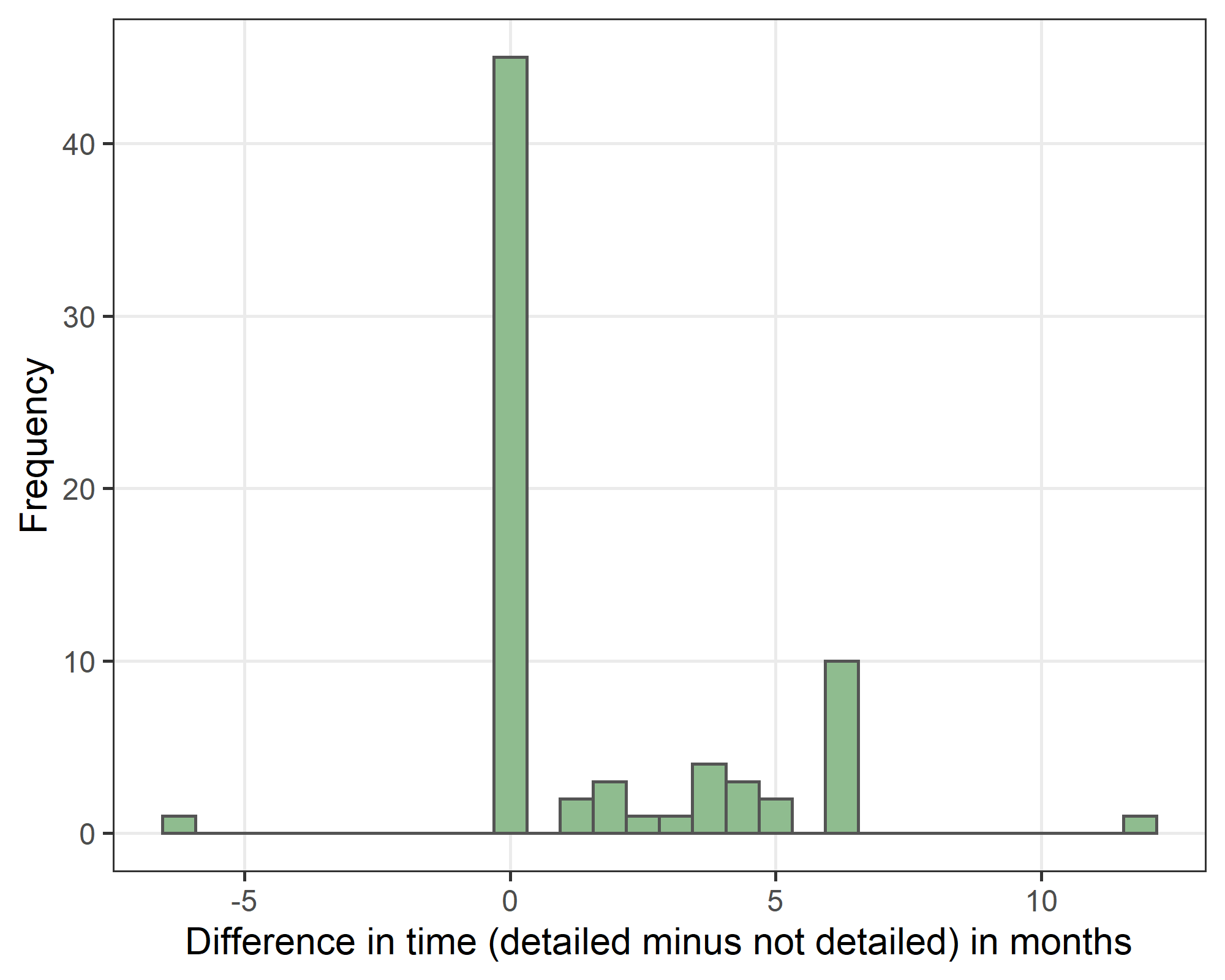


The histogram shows many people treat the applicants equally, but whenever there is a difference there is almost always a greater time off given to those who gave the details.

A non-parametric bootstrap test of the paired difference gives a mean of 1.6 months with a 95% confidence interval of 1.0 to 2.3 months. So there is evidence that those giving their medical details get a larger adjustment from peer reviewers.

In a sensitivity analysis we assumed that those who left the slider at the default position of six meant to answer 6. A bootstrap test of the paired difference gives a mean of 1.2 months with a 95% confidence interval of 0.9 to 1.5 months

## Comfort/discomfort with reading others medical sections

*Imagine you are reading a career disruption section about somebody that you know. This is not a close colleague, as conflict of interest rules would mean you could not review their application. How comfortable are you reading personal social and/or medical details as part of the peer review process?*

| **Response** | **Non-random** | **Random** | **Total** |
| --- | --- | --- | --- |
| Extremely comfortable | 20 (18) | 27 (24) | 47 (21) |
| Somewhat comfortable | 30 (27) | 26 (23) | 56 (25) |
| Neither comfortable nor uncomfortable | 12 (11) | 22 (19) | 34 (15) |
| Somewhat uncomfortable | 38 (34) | 34 (30) | 72 (32) |
| Extremely uncomfortable | 12 (11) | 5 (4) | 17 (8) |


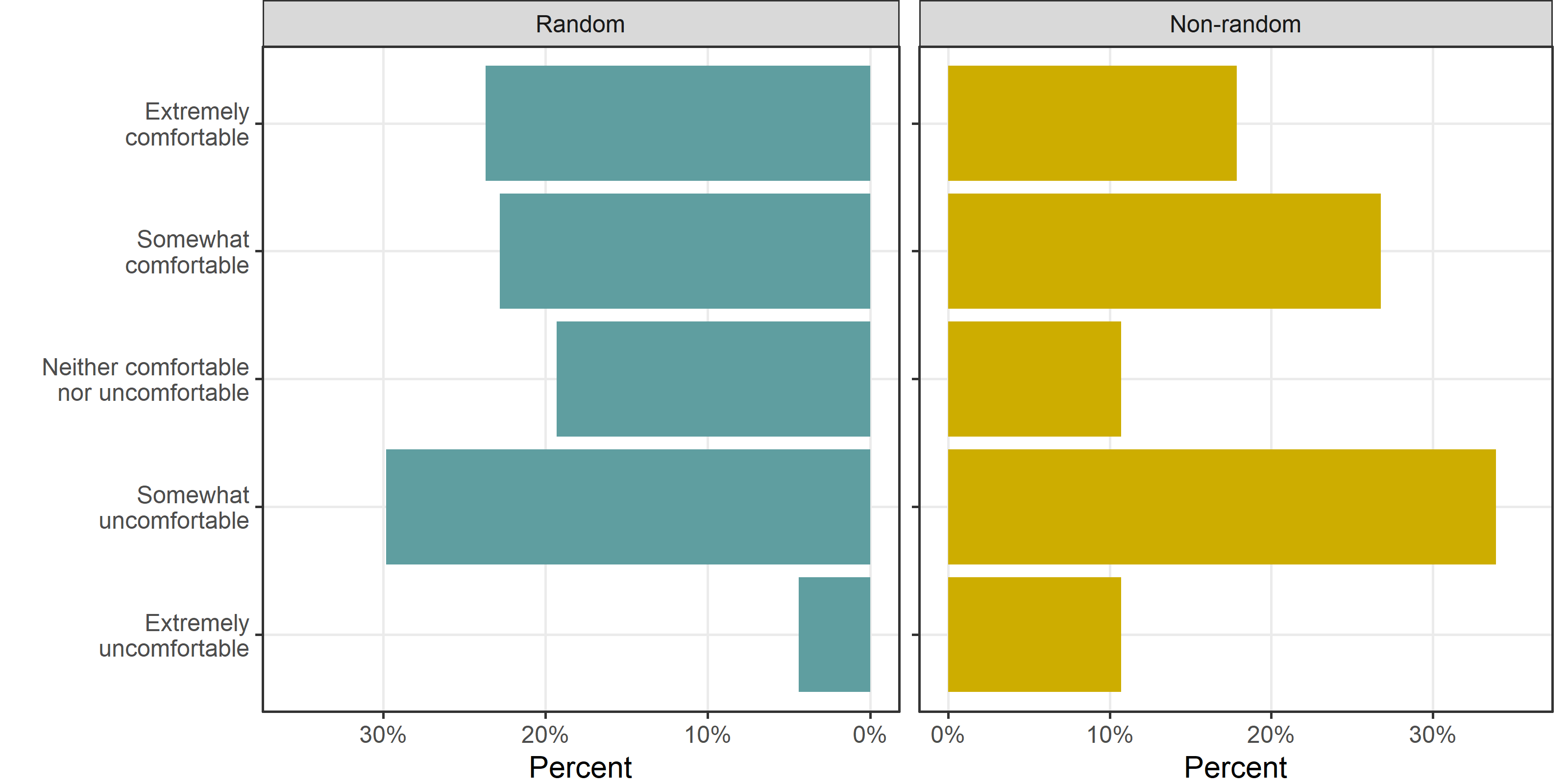


##### Confidence interval for two uncomfortable categories combined.

| **Response** | **n** | **p** | **lower** | **upper** |
| --- | --- | --- | --- | --- |
| Comfortable | 53 | 46 | 38 | 57 |
| Neutral | 22 | 19 | 11 | 30 |
| Uncomfortable | 39 | 34 | 25 | 45 |

# Independent panel for assessing career disruption

## *How do you think career disruption sections in NHMRC applications should be assessed?*

| **Response** | **Non-random** | **Random** | **Total** |
| --- | --- | --- | --- |
| Scientific panel (current system) | 18 (16) | 30 (26) | 48 (21) |
| Independent medical panel (new system) | 49 (44) | 40 (35) | 89 (39) |
| Not sure | 36 (32) | 32 (28) | 68 (30) |
| Other | 9 (8) | 12 (11) | 21 (9) |


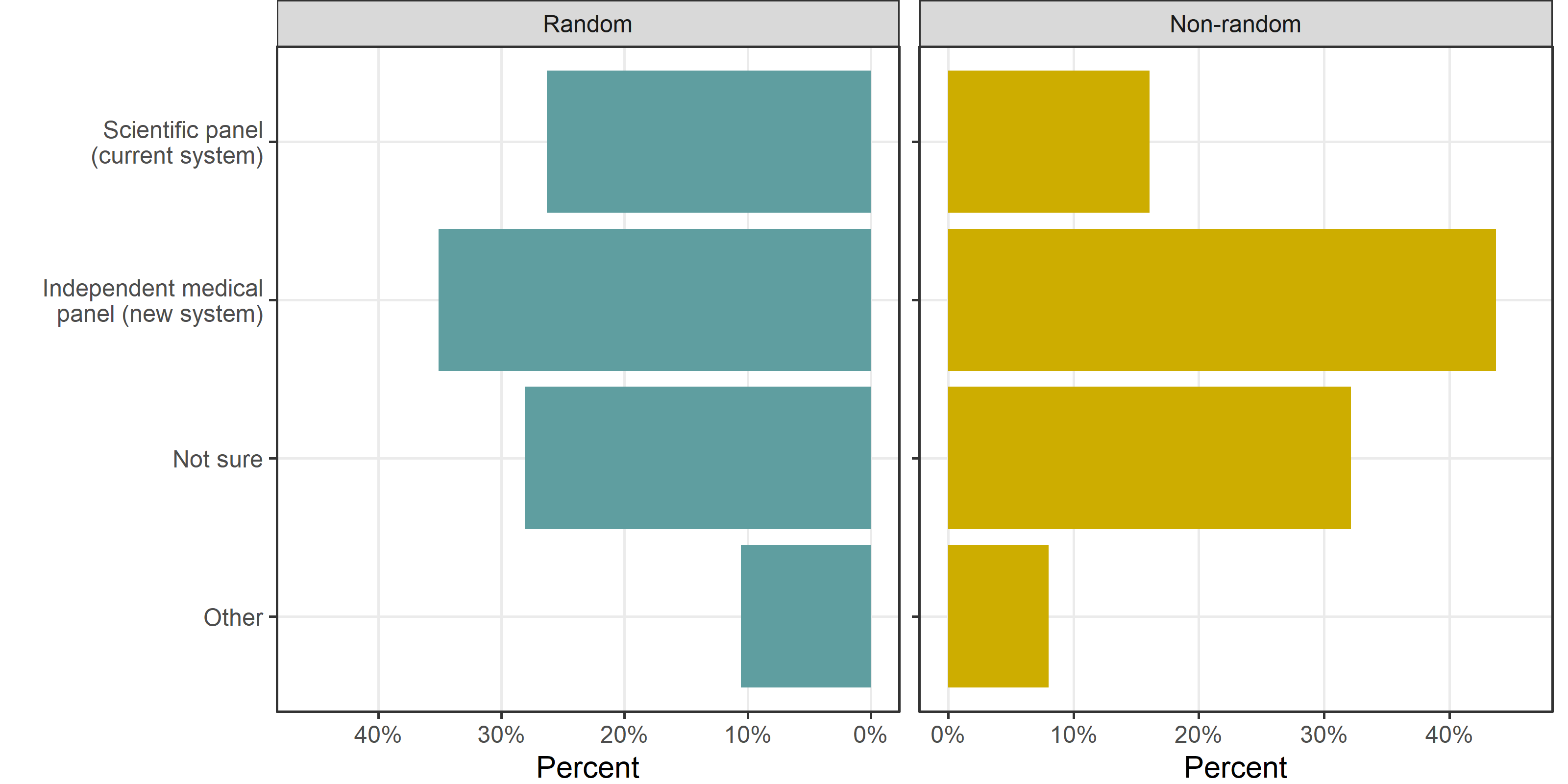


##### Confidence intervals for random sample:

| **Response** | **n** | **p** | **lower** | **upper** |
| --- | --- | --- | --- | --- |
| Scientific panel (current system) | 30 | 26 | 18 | 37 |
| Independent medical panel (new system) | 40 | 35 | 26 | 46 |
| Not sure | 32 | 28 | 19 | 39 |
| Other | 12 | 11 | 2 | 21 |

# Respondent characteristics

## Broad research area

| **Response** | **Non-random** | **Random** | **Total** |
| --- | --- | --- | --- |
| Basic Science | 40 (36) | 38 (33) | 78 (35) |
| Clinical Medicine and Science | 25 (22) | 36 (32) | 61 (27) |
| Public Health | 23 (21) | 23 (20) | 46 (20) |
| Health Services Research | 24 (21) | 17 (15) | 41 (18) |


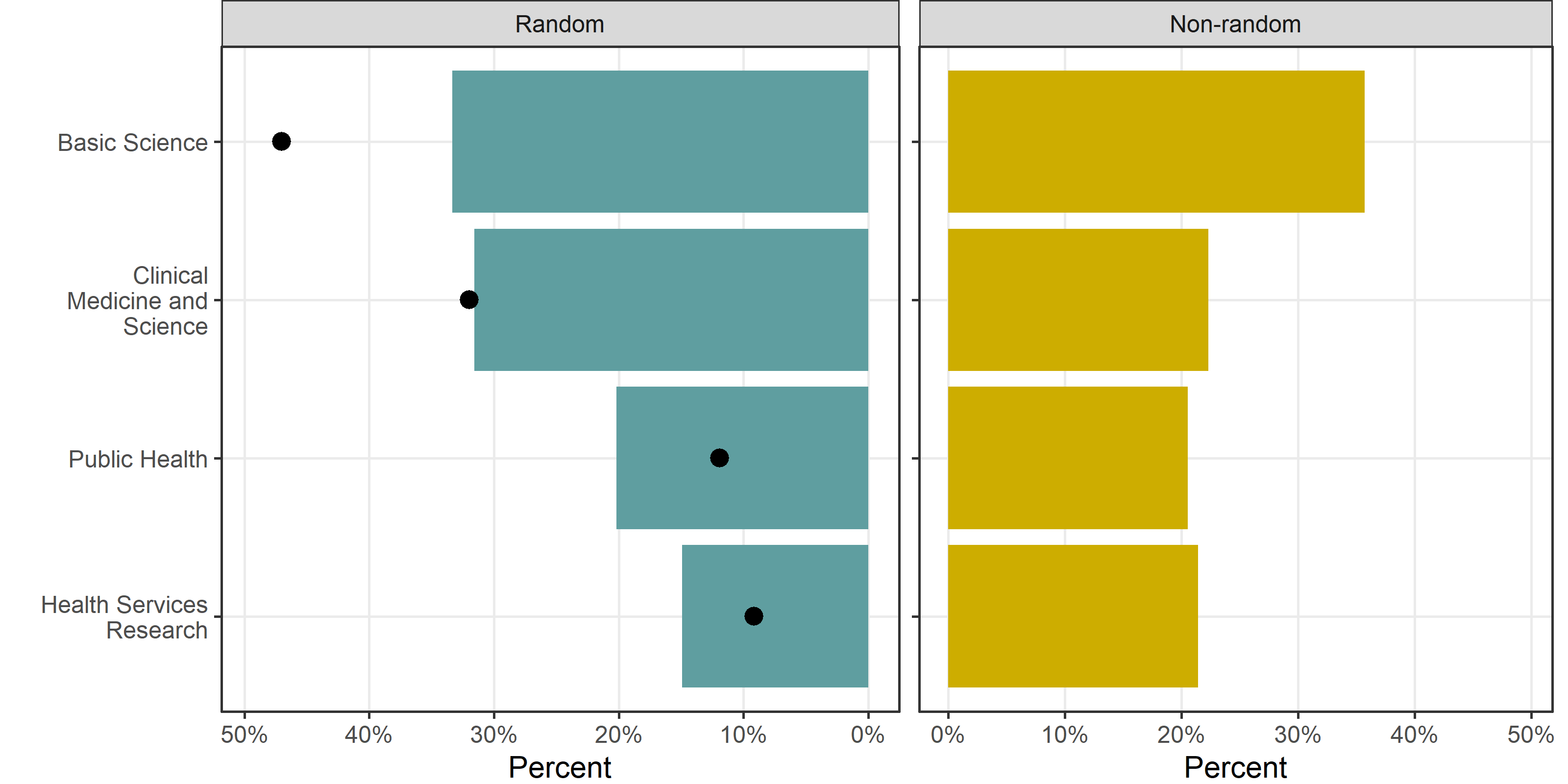


The black dots show the percentage of applications from the four fields from 2019 data. Our response numbers have under-represented Basic Science and over-represented Public Health and Health Services Research.

## Gender

| **Response** | **Non-random** | **Random** | **Total** |
| --- | --- | --- | --- |
| Male | 20 (18) | 52 (46) | 72 (32) |
| Female | 89 (79) | 60 (53) | 149 (66) |
| Non-binary / third gender | 2 (2) | 1 (1) | 3 (1) |
| Missing | 1 (1) | 1 (1) | 2 (1) |


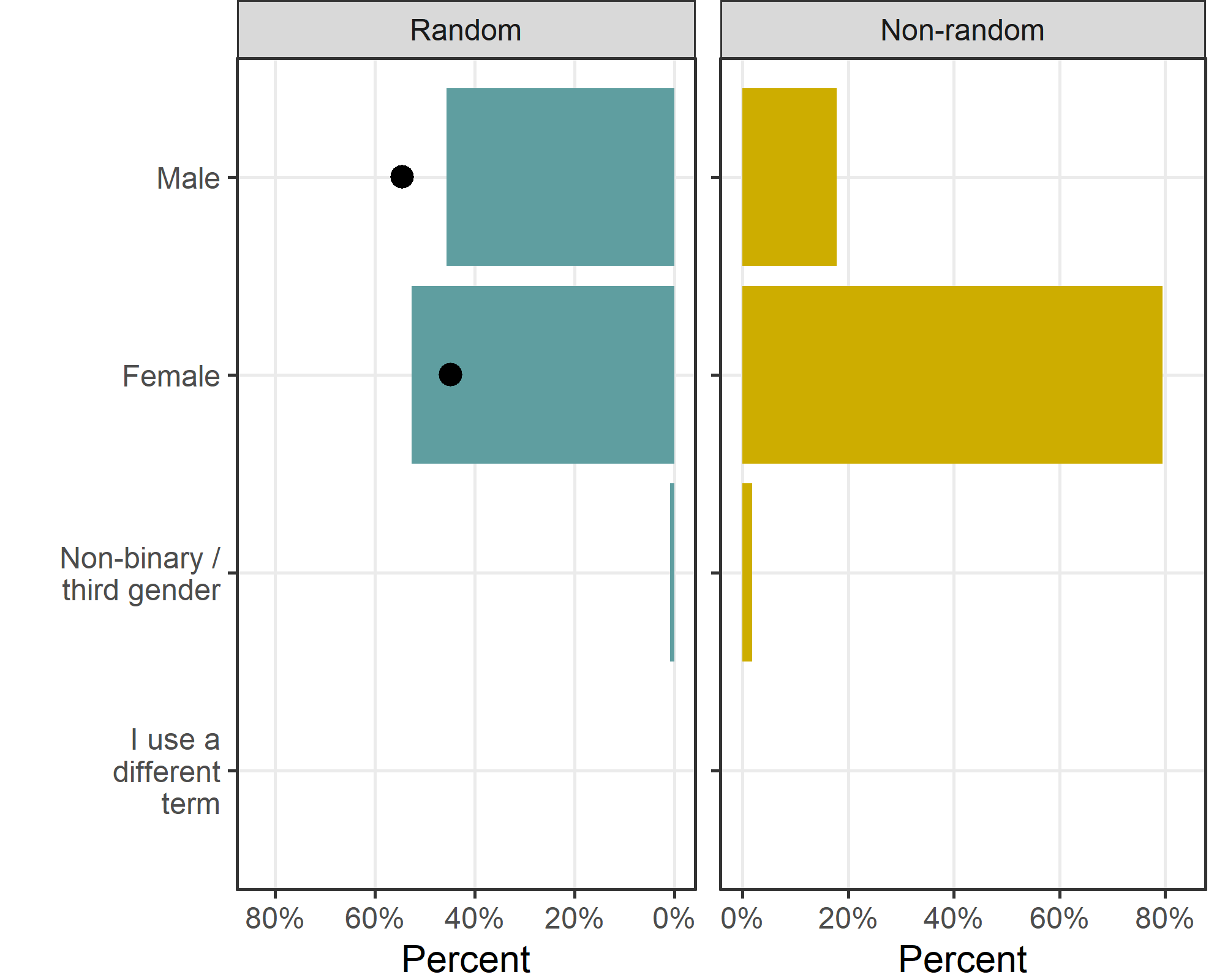


There is a big difference in gender between the random and non-random samples, with many more women in the non-random sample.

The black dots show the percentage of male and female applicants from 2020 data.

## Approximate number of years in health and medical research

| **Response** | **Non-random** | **Random** | **Total** |
| --- | --- | --- | --- |
| 5 years or less | 17 (15) | 26 (23) | 43 (19) |
| 6 to 10 years | 31 (28) | 30 (26) | 61 (27) |
| 11 to 15 years | 35 (31) | 24 (21) | 59 (26) |
| 16 to 20 years | 19 (17) | 16 (14) | 35 (15) |
| 21 years or more | 10 (9) | 18 (16) | 28 (12) |


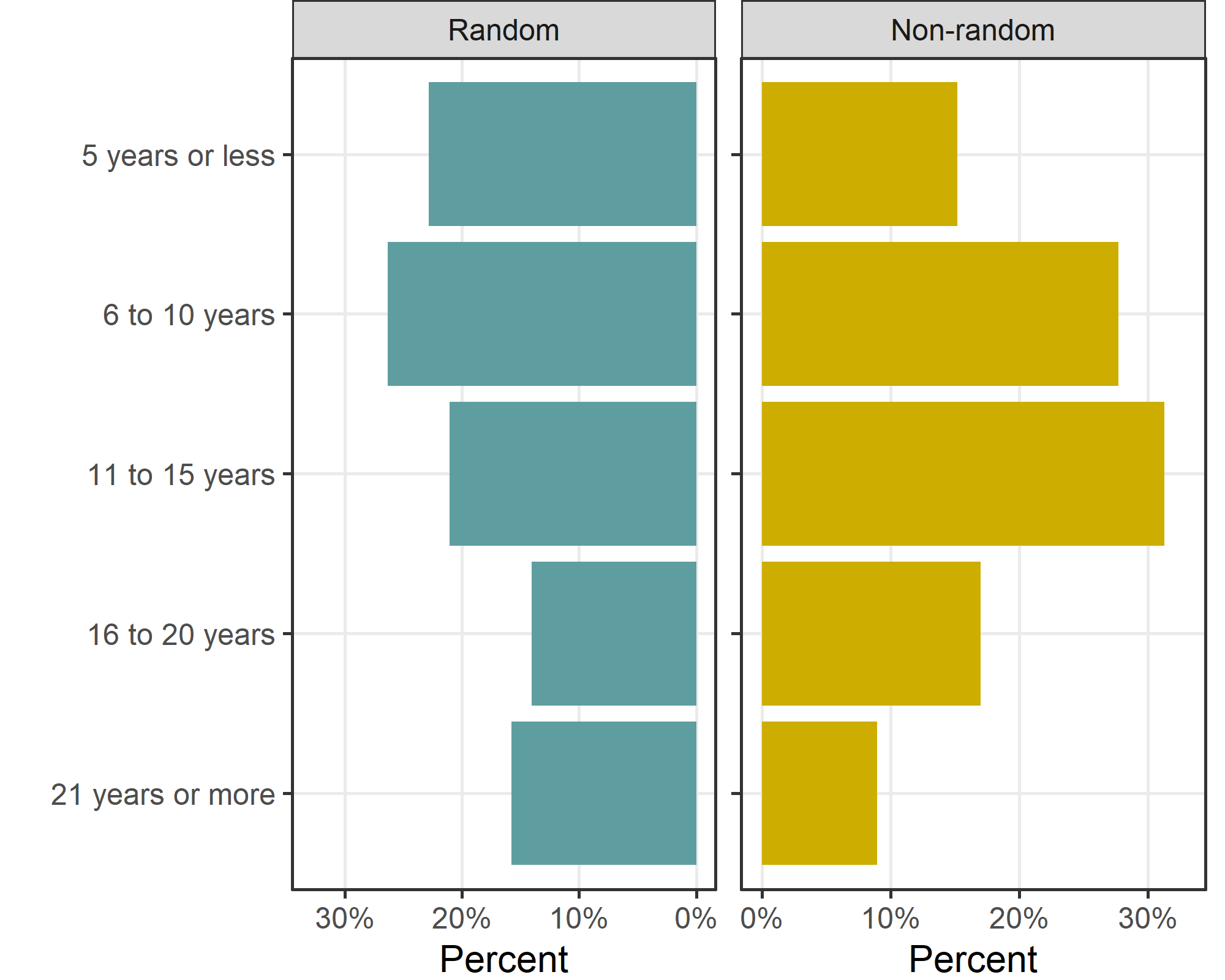


# Differences by gender

In this section we look for differences in the responses by gender. We examine a selection of the survey questions to assess whether women were more or less likely to agree. We used ordinal regression for responses on an ordinal scale, such as disagree, neutral and agree. We used logistic regression for responses on an nominal scale and selected a particular response, e.g., support for the medical panel.


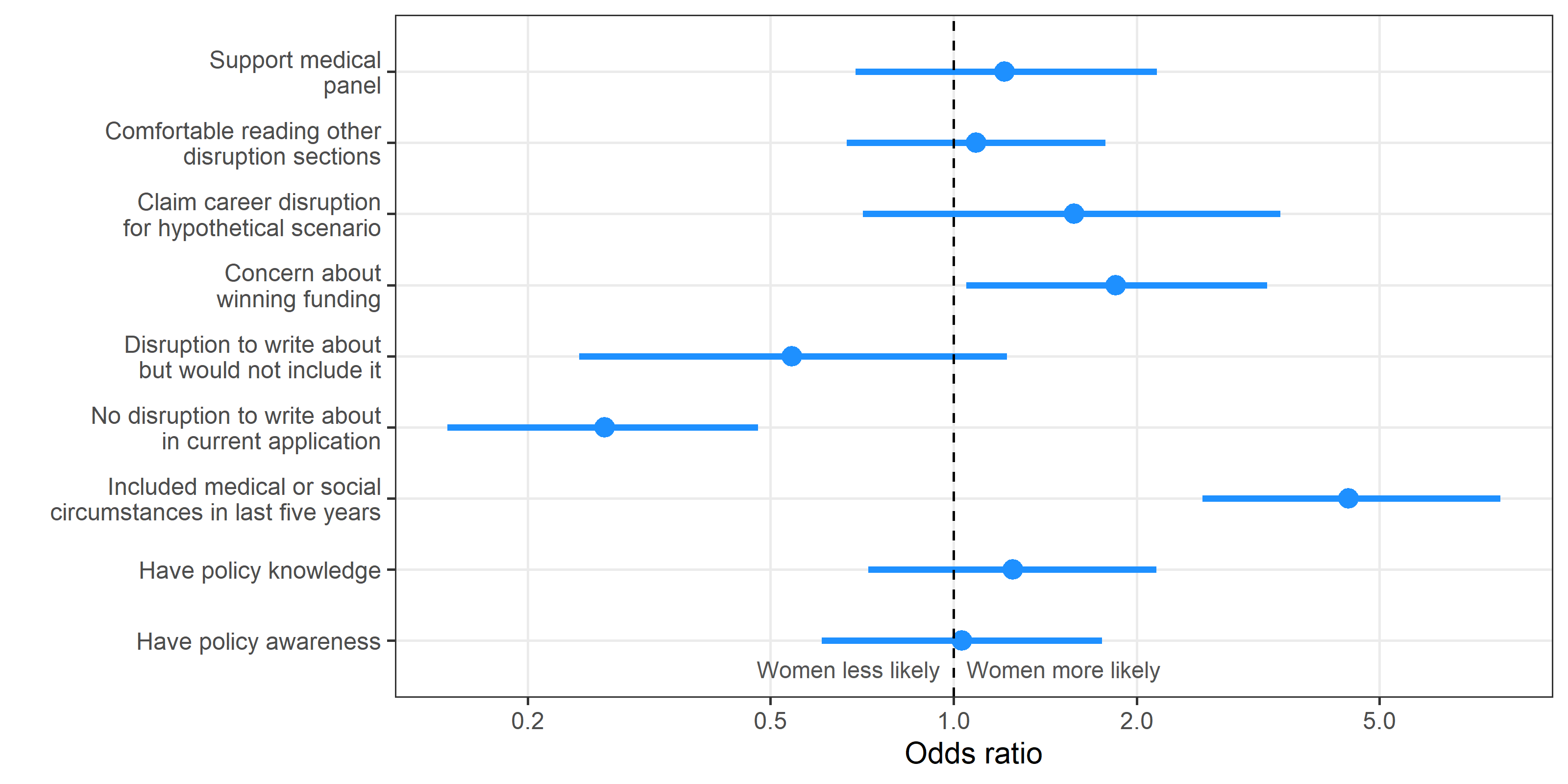


# Survey response results

In this section we examine meta-data on the survey including the completeness and time taken.

## Flow diagram (random sample)

This plot shows the flow of respondents for the random sample.


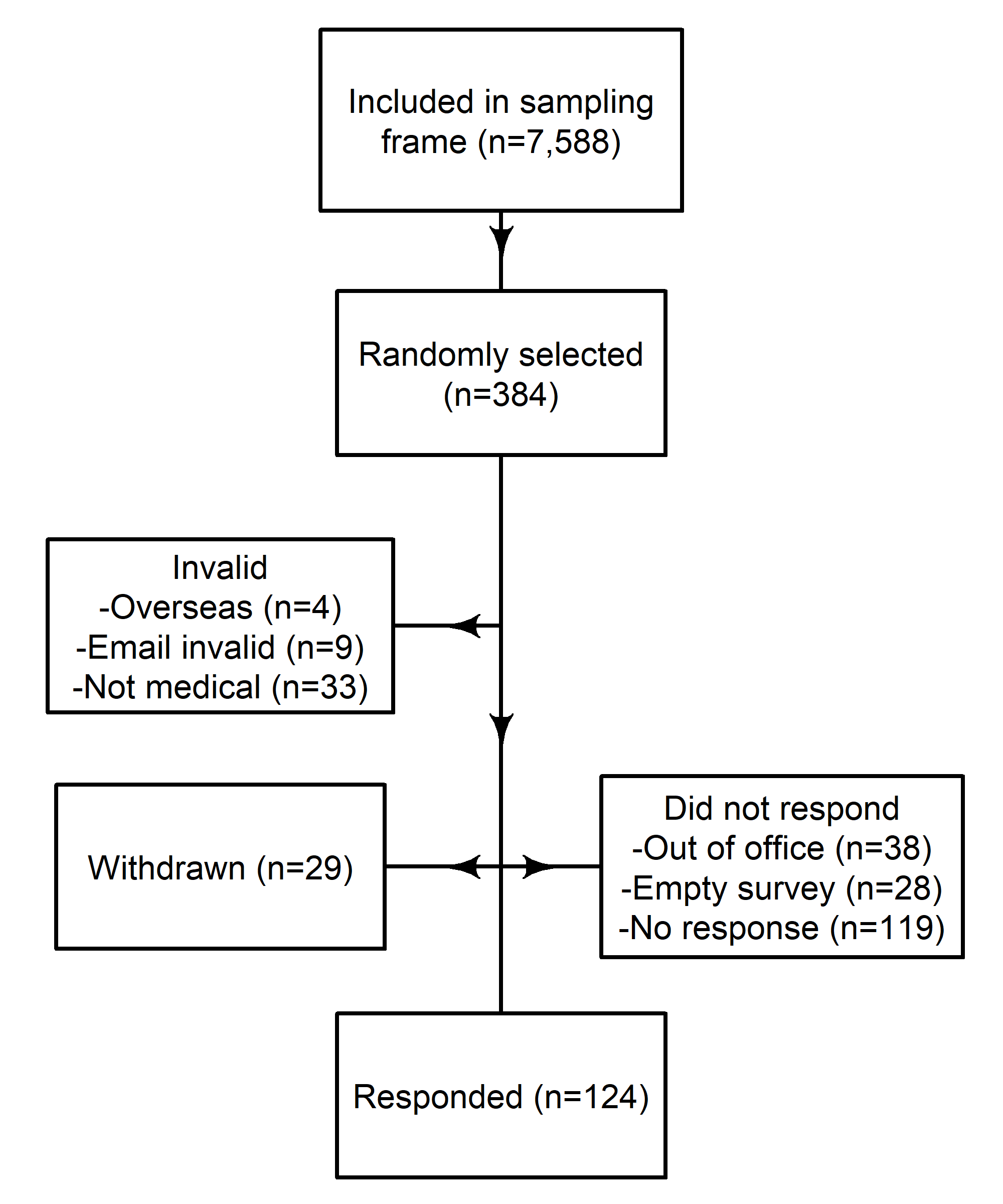


## Dates


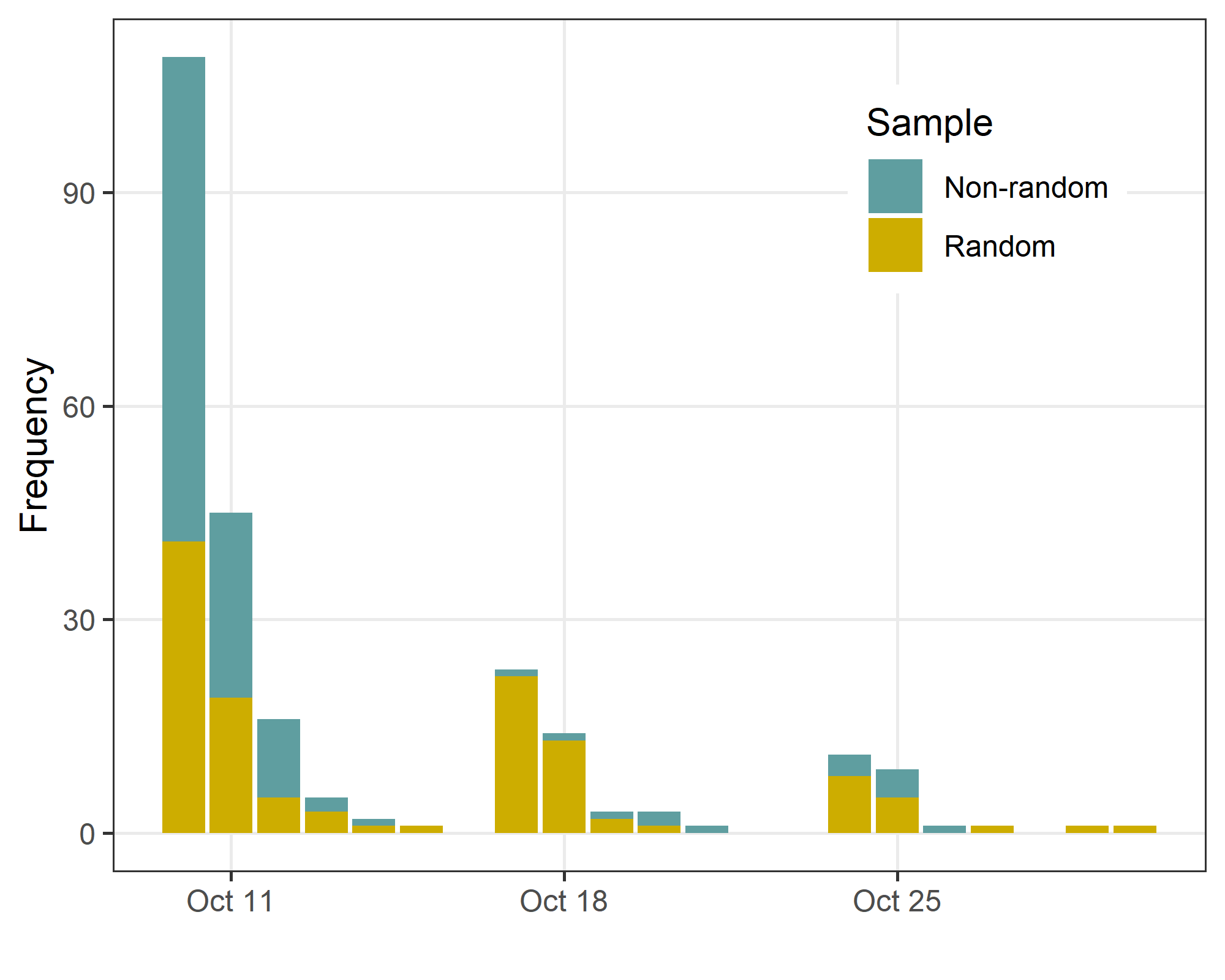


The survey was launched on October 10. Reminders were sent 1 and 2 weeks later to non-responders and incomplete responders in the random sample.

## Survey progress as a percent

The table below shows the survey progress as a percent, with the results grouped into three categories.

| **Response** | **Non-random** | **Random** | **Total** |
| --- | --- | --- | --- |
| (5,50] | 7 (6) | 7 (6) | 14 (6) |
| (50,75] | 2 (2) | 3 (2) | 5 (2) |
| (75,100] | 113 (93) | 114 (92) | 227 (92) |

## Time taken

Time taken in minutes to complete the survey. Cells show the median and inter-quartile range.

| **sample** | **Median (IQR)** |
| --- | --- |
| Non-random | 8 (6 to 13) |
| Random | 8 (6 to 15) |
| Total | 8 (6 to 14) |

## Numerical summary of optional text comments

| **section** | **labels** | **Non-random** | **Random** |
| --- | --- | --- | --- |
| 3 | Do you have any comments on this section? | 27 / 27 | 23 / 24 |
| 4 | Any other thoughts around medical or social circumstances you would not include | 35 / 25 | 21 / 33 |
| 4 | Any other thoughts around medical or social circumstances you would include | 17 / 29 | 16 / 30 |
| 4 | Do you have any comments on this section? | 15 / 40 | 7 / 32 |
| 6 | Any other thoughts around what you would not include | 1 / 7 | 3 / 31 |
| 6 | Any other thoughts around what you would include | 23 / 20 | 20 / 21 |
| 6 | Do you have any comments on this section? | 19 / 27 | 18 / 30 |
| 7 | Do you have any comments on this section? | 25 / 36 | 31 / 37 |
| 8 | Do you have any comments on this section? | 46 / 34 | 25 / 50 |
| 9 | Do you have any comments on this survey? | 24 / 20 | 18 / 27 |

The total number of comments was 414.

The table shows the number of comments and the median number of words.

There were more comments from the non-random sample who were 42% more likely to provide a comment, with a 95% confidence interval from -2% to 106%.

# Missing data

This section examines the item-missing data of missing responses to each question. The plot below shows what responses were missing, excluding optional comments. The questions are ordered according to the survey.

The four panels show the missing data by sample and by their response to the question in section 5 about whether they would write anything about career disruption for the hypothetical scenario, as these two aspects control what responses should have been completed.


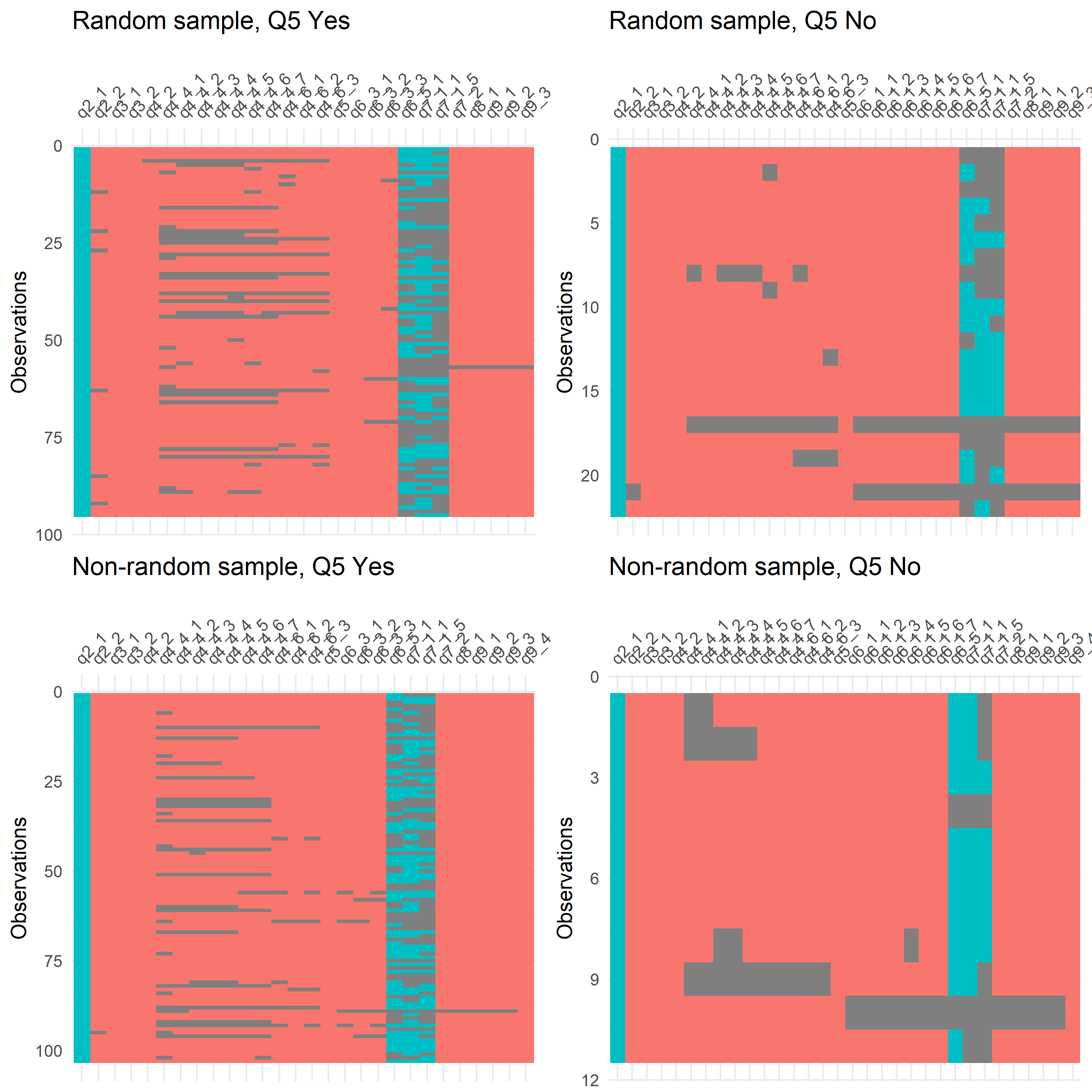


### Counts of missing data by question

| **Question** | **Maximum possible** | **Number missing** | **Percent missing** |
| --- | --- | --- | --- |
| How many NHMRC applications have you been part of in the last 5 years as a chief investigator? Please include all types, so Projects Grants, Fellowships, Partnerships, Ce | 231 | 0 | 0 |
| How aware are you of the current NHMRC policies around documenting career disruption in grant applications? | 231 | 0 | 0 |
| I understand what medical or social circumstances could be mentioned in an NHMRC career disruption section. | 231 | 0 | 0 |
| In this section, please consider the following hypothetical scenario and think about what you would write in the career disruption section. Imagine you had missed six mon | 231 | 0 | 0 |
| We would like to know your email. This is only to know if you were part of our random sample who we emailed to take part; spam filters may have trapped some invites. Your | 114 | 0 | 0 |
| Would you write anything in your application about disruption to your research because of medical or social circumstances in the last five years? | 231 | 1 | 0 |
| Share all my personal details about the issue | 198 | 4 | 2 |
| Imagine you are reading a career disruption section about somebody that you know. This is not a close colleague, as conflict of interest rules would mean you could not re | 231 | 5 | 2 |
| Career disruption sections are currently assessed by scientific panels, who judge how a personal or medical issue has impacted on the researcher’s track record. An altern | 231 | 5 | 2 |
| Lastly, we'd like to know some basic information about you. What is your broad research area? | 231 | 5 | 2 |
| Gender | 231 | 5 | 2 |
| Approximate number of years in health and medical research. Answer in terms of working years, e.g., if 6 years of working half-time then answer 3 years ("5 years or less" | 231 | 5 | 2 |
| Partially share my personal details about the issue | 198 | 6 | 3 |
| How often in those applications did you include medical or social circumstance that disrupted your research? | 231 | 8 | 3 |
| Not share any personal details | 198 | 9 | 5 |
| Share only the basic information needed to convey the issue | 231 | 16 | 7 |
| This issue should only have a minor impact on my research | 33 | 3 | 9 |
| Because sharing this information could reduce my chances of winning funding | 33 | 3 | 9 |
| I would not want to share personal details with my co-investigators | 33 | 3 | 9 |
| I would not want to share personal details with my peer reviewers | 33 | 3 | 9 |
| I am unsure of how to use the “career disruption” and/or “career context” sections in NHMRC applications. | 33 | 3 | 9 |
| Career disruption is just part of the lottery of life and should not be adjusted for. | 33 | 3 | 9 |
| Share personal details about the issue | 231 | 23 | 10 |
| Not share any details about the issue, but instead state how much time you’ve lost to research | 231 | 23 | 10 |
| I would be worried about my reputation | 33 | 4 | 12 |
| Career disruption is just part of the lottery of life and should not be adjusted for | 231 | 34 | 15 |
| I am unsure of how to use the “career disruption” and/or “career context” sections in NHMRC applications | 231 | 40 | 17 |
| I do not want to share personal details with peer reviewers | 231 | 43 | 19 |
| I am concerned that sharing this information would reduce my chances of winning funding | 231 | 44 | 19 |
| I do not want to share personal details with my co-investigators | 231 | 44 | 19 |
| I am worried about my reputation | 231 | 44 | 19 |
| My circumstances have had only a minor impact on my research | 231 | 58 | 25 |
| Imagine you are comparing two NHMRC fellowship applications for which both applicants have stated that they lost 6 months of research time in the last 5 years due to car | 231 | 102 | 44 |
| How much time away from research would you include in your career disruption section because of this issue? - Months | 231 | 113 | 49 |
| Imagine you are comparing two NHMRC fellowship applications for which both applicants have stated that they lost 6 months of research time in the last 5 years due to car | 231 | 148 | 64 |

The three questions with the most amount of missing were the three slider questions:

- *How much time away from research would you include in your career disruption section because of this issue?* q6_5_1
- *How much time would you adjust for for each applicant - Applicant who gave no details* q7_1_1
- *How much time would you adjust for for each applicant - Applicant who explained their medical issue in detail* q7_1_5

This is because respondents had to move each slider at least slightly for the question to count as answered. Hence we did a sensitivity analysis where we imputed 6 for missing results.

All other questions were reasonably well completed.
